# Supplementary material for: Large-scale exploration of whole-brain structural connectivity in anorexia nervosa: alterations in the connectivity of frontal and subcortical networks
Source: Biol Psychiatry Cogn Neurosci Neuroimaging. Author manuscript; Available in PMC 2024 Apr 30. (PMC11060509; doi:10.1016/j.bpsc.2022.06.002)
Supplement: Supplementary material [file NIHMS1974045-supplement-Supplementary_material.pdf]

Large-Scale Exploration of Whole-Brain Structural Connectivity in Anorexia Nervosa: Alterations in the Connectivity of Frontal and Subcortical Networks

Supplementary Information

Table of Contents

MRI Scan Parameters..........2
Participant characteristics by study and diagnosis..........3
Methodological details..........4
Probabilistic tractography..........4
Network-based statistic..........4
Node-based analyses..........4
Details of component connectivity by study and diagnosis..........5
Full results of node analyses..........6
Unharmonized data analyses..........18
Subtype analyses..........23
Subsample analyses..........23
Supplementary References..........24

MRI Scan Parameters

Table S1: MRI scan parameters across the five studies

Study	Scanner model	T1 structural MRI scan parameters	Diffusion-weighted MRI scan parameters
Study 1	1.5 T Philips Intera MRI system with 8-channel head coil	Sequence: T1weighted 3D spoiled gradient recall (SPGR)<br>TR: 2.5 ms<br>TE: 3.7 ms<br>flip angle: 30°<br>FOV: 256 mm<br>128 slices<br>voxel size: 1 × 1 × 1 mm	Diffusion series: two images with minimal diffusion weighting, 16 images with diffusion weighting along non-collinear directions (b-value=800 sm−2)<br>Runs: 2<br>TR: 10,58.6 ms<br>TE:7.0 ms<br>70 slices<br>voxel size: 2 × 2 × 2 mm
Study 2	3.0T Phillips MRI system with 8-channel head coil.	Sequence: T1weighted Magnetization Prepared Rapid Acquisition Gradient Echo (MP-RAGE)<br>TR: 6.5 ms<br>TE: 3.0 ms<br>flip angle: 8°<br>FOV: 256 mm<br>165 slices<br>voxel size: 1 × 1 × 1 mm	Diffusion series: one image with minimal diffusion weighting, 56 images with diffusion weighting along non-collinear directions (b-value=800 sm^{-2})<br>Runs: 1<br>TR: 775.5 ms<br>TE: 6.8 ms<br>75 slices<br>voxel size: 2 × 2 × 2 mm
Study 3	GE Signa 3T MRI system with 32-channel head coil	Sequence: T1weighted 3D (SPGR) – BRAVO sequence<br>flip angle: 11°<br>FOV: 230 mm<br>voxel size: 0.9 × 0.9 × 0.9	Diffusion series: three images with minimal diffusion weighting, 25 images with diffusion weighting along non-collinear directions (b-value=1000 sm−2)<br>Runs: 1<br>TR: 850 ms<br>TE: 8.23 ms<br>75 slices<br>voxel size: 0.94 × 0.94 × 2.5 mm
Study 4	GE Signa 3T MRI system with 32-channel head coil	Sequence: T1weighted 3D (SPGR) – BRAVO sequence<br>flip angle: 12°<br>FOV: 256 mm<br>voxel size: 1mm × 1mm × 1mm	Diffusion series: two images with minimal diffusion weighting, 56 images with diffusion weighting along non-collinear directions (b-value=800 sm^{-2})<br>TR: 900 ms<br>TE: minimum<br>75 slices<br>voxel size: 1.5 x 1.5 x 2.0 mm
Study 5 scanner 1	GE Signa 3T MRI system with 32-channel head coil	Sequence: T1weighted 3D (SPGR) – BRAVO sequence<br>flip angle: 12°<br>FOV: 256 mm<br>voxel size: 1mm × 1mm × 1mm	Diffusion series: two images with minimal diffusion weighting, 56 images with diffusion weighting along non-collinear directions (b-value=800 sm−2)<br>TR: 900 ms<br>TE: minimum<br>75 slices<br>Runs: 1<br>voxel size: 1.5 x 1.5 x 2.0 mm
Study 5 scanner 2	GE Signa 3T MRI system with 48-channel head coil	Sequence: T1weighted 3D (SPGR) – BRAVO sequence<br>flip angle: 12°<br>FOV: 256 mm<br>voxel size: 1mm × 1mm × 1mm	Diffusion series: two images with minimal diffusion weighting, 56 images with diffusion weighting along non-collinear directions (b-value=800 sm^{-2})<br>Runs: 1<br>TR: 900 ms<br>TE: minimum<br>75 slices<br>voxel size: 1.5 x 1.5 x 2.0 mm

Participant characteristics by study and diagnosis

Table S2: Participant characteristics by study and diagnosis

	Study 1		Study 2		Study 3		Study 4		Study 5	
	HC (N=20)	AN (N=23)	HC (N=25)	AN (N=26)	HC (N=13)	AN (N=16)	HC (N=35)	AN (N=30)	HC (N=26)	AN (N=52)
	Mean ± SD (range)	Mean ± SD (range)	Mean ± SD (range)	Mean ± SD (range)	Mean ± SD (range)	Mean ± SD (range)	Mean ± SD (range)	Mean ± SD (range)	Mean ± SD (range)	Mean ± SD (range)
Age (years)	20.7 ± 2.98 (16, 25)	19.26 ± 2.49 (16, 25)	19.36 ± 3.01 (15, 24)	19.35 ± 3.49 (14, 26)	22.08 ± 2.47 (18,2 7)	26.88 ± 6.93 (16, 39)	25.86 ± 5.35 (19, 39)	26.93 ± 6.07 (17, 40)	15.88 ± 1.58 (12, 18)	15.38 ± 1.62 (12, 18)
BMI (kg/m^2)[a]	21.47 ± 1.67 (19.9, 24.3) (N=13)	17.36 ± 0.92 (15.65, 18.24) (N=8)	21.24 ± 1.61 (18.9, 23.4) (N=9)	15.46 ± 2.25 (12.51, 18.12) (N=13)	21.51 ± 2.33 (19.1, 24.9) (N=11)	15.32 ± 1.66 (11.72, 17.74) (N=14)	21 ± 1.60 (18.7, 25.0) (N=32)	16.00 ± 1.65 (13.52, 18.73) (N=25)	NA (N=0)	NA (N=0)
BMI percentile (%)[b]	35.26 ± 16.7 (19.4, 70.1) (N=7)	3.92 ± 7.42 (0, 28.6) (N=15)	50.72 ± 19.23 (21.1, 86.2) (N=16)	7.64± 6.77 (0, 21.2) (N=13)	17.75 ± 16.48 (6.1, 29.4) (N=2)	7.65 ± 3.32 (5.3, 10.0) (N=2)	22.13 ± 5.45 (16.3, 27.4) (N=3)	7.62 ± 7.11 (0.7, 17.8) (N=5)	54.54 ± 24.67 (13.3, 87.4) (N=26)	9.56 ± 10.46 (0, 39.6) (N=52)
EDEQ Global score	0.09 ± 0.14 (0, 0.45)	3.99 ± 1.36 (1.46, 5.65)	0.19 ± 0.2 (0, 0.75)	4.14 ± 1.41 (0.30, 5.6)	0.09 ± 0.09 (0, 0.25)	4.43 ± 1.14 (1.38, 5.65)	0.35 ± 0.48 (0, 0.2.09)	4.27 ± 1.5 (0.03, 5.80)	0.42 ± 0.49 (0, 0.2.17)	3.39 ± 1.68 (0, 6)
Illness Duration (months)	NA ± NA	52.48 ± 37.72 (3, 120)	NA ± NA	38.19 ± 28.42 (8, 120)	NA ± NA	144.5 ± 82.34 (12, 278)	NA ± NA	118.53 ± 79.39 (12, 324)	NA ± NA	8.51 ± 11.8 (0, 59)
Mean framewise displacement across DWI series	0.2 ± 0.1	0.26 ± 0.16	0.11 ± 0.13	0.05 ± 0.07	0.32 ± 0.04	0.33 ± 0.03	0.12 ± 0.05	0.11 ± 0.04	0.16 ± 0.07	0.2 ± 0.17

[a] BMI information presented for participants over 19
[b] BMI percentile information presented for participants 19 and under
BMI: Body mass index; EDEQ: Eating disorder examination questionnaire

Methodological details

Probabilistic tractography

Response functions of white matter, grey matter and CSF were derived with the dhollander algorithm (1), using the dMRI signal and the five-tissue type image generated from the structural MRI scan. Next, the response functions of white matter fibres were estimated in the voxels with the 300 greatest fractional anisotropy values (indicative of more directed diffusion). Using the dMRI signal and the calculated response functions, the underlying distribution of fibers in each voxel was recovered using multi-shell multi-tissue constrained spherical deconvolution (2), with default maximum spherical harmonic degree parameters. This approach allows dMRI signal resulting from white matter and CSF components to be distinguished, preventing CSF contaminating estimates of white matter fiber orientation distributions. White matter fiber orientation distributions were normalized, to remove residual inhomogeneity in intensities. Seed points for streamlines were determined dynamically. Seeding continued until 20,000,000 streamlines between 10 and 250 mm were generated. Streamline step size was 0.625 mm; the maximum angle between successive steps was 22.5 degrees.

The iFOD2 (3) tracking algorithm determines the probability of candidate streamline paths by sampling fiber orientation distributions along the entire path. More probable, and thus reconstructed, streamlines are generally those where fiber orientation distribution amplitudes along the path are large. The tractography was anatomically constrained, with the five-tissue type image used as a biological prior to guide streamline termination and acceptance (4). Streamlines were re-tracked if a poor termination was encountered, and cropped at the grey matter white matter interface.

Network-based statistic

To complete the network-based statistic analysis, first tracts differing in streamline count between groups at a t-score threshold of greater than 3.1 (corresponding to a p-value of 0.001) are identified. Next, components, or sets of connected tracts meeting the t-score criteria, are identified. The null distribution of the maximum component size is empirically estimated using a permutation approach. P-values for the hypothesis that observed components (i.e., those identified in the unpermuted data) are larger than expected should there be no between-group difference in subnetwork connectivity can then be derived. 10,000 permutations were specified, and identified components were significant at a family-wise error corrected rate of $p < 0.05$.

Node-based analyses

Strength is calculated as the total number of connections (streamlines) of each node. To calculate efficiency, the inverse of the shortest path length between all nodes directly connected to the node in question was determined and averaged. Path length was defined as the inverse of streamline count. The contribution of a given path to efficiency calculations was proportionate to the streamline count between the path nodes and the node for which efficiency was calculated (i.e., the average was weighted).

Details of component connectivity by study and diagnosis

Table S3: Connectivity in subcortical and cortical components by study and diagnosis

	Study 1		Study 2		Study 3		Study 4		Study 5		Full sample	
	HC (N=20)	AN (N=23)	HC (N=25)	AN (N=26)	HC (N=13)	AN (N=16)	HC (N=35)	AN (N=30)	HC (N=26)	AN (N=52)	HC (N=119)	AN (N=147)
	Mean (SD)	Mean (SD)	Mean (SD)	Mean (SD)	Mean (SD)	Mean (SD)	Mean (SD)	Mean (SD)	Mean (SD)	Mean (SD)	Mean (SD)	Mean (SD)
Subcortical component connectivity	6416.44 (1044.80)	5926.73 (779.30)	6490.86 (933.36)	5937.17 (751.08)	6775.51 (1710.19)	5481.15 (677.18)	6387.86 (832.13)	5637.40 (804.14)	6536.10 (793.97)	5741.32 (832.15)	6849.04 (998.46)	5755.44 (791.98)
Cortical component connectivity	131.27 (33.42)	202.50 (70.40)	155.28 (66.00)	186.54 (49.70)	145.03 (72.41)	209.88 (67.63)	164.51 (62.49)	188.10 (71.11)	133.12 (45.27)	182.76 (64.36)	148.00 (57.86)	190.56 (64.66)
Connectivity defined as average streamline count in component connections												

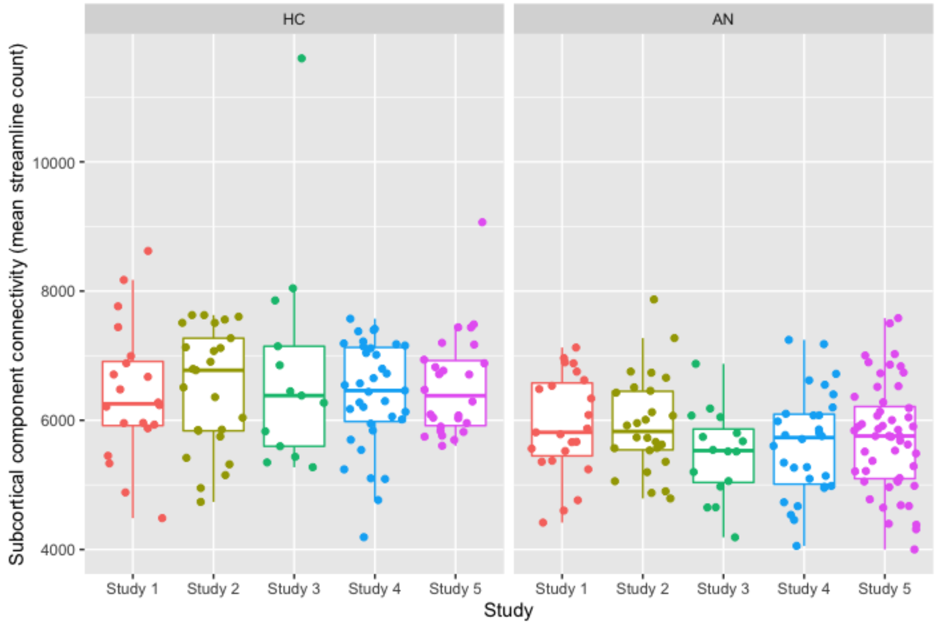

Figure S1: Distribution of subcortical connectivity values across studies and diagnostic groups

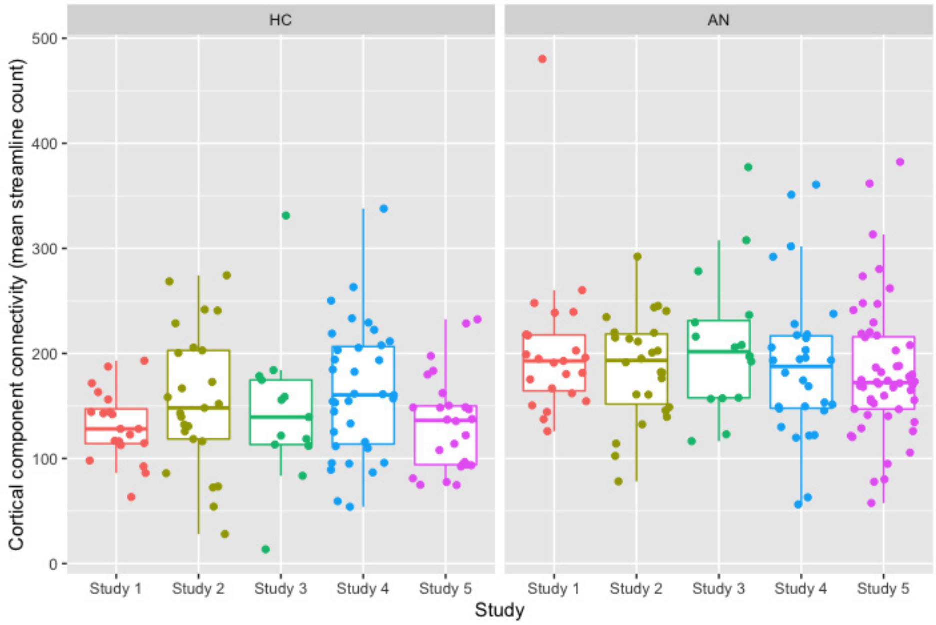

Figure S2: Distribution of cortical connectivity values across studies and diagnostic groups

Full results of node analyses

Table S4: Outcomes of between-group analyses of node strength

	Strength			
Node	HC	AN	Effect of Diagnosis	
	Mean (SD)	Mean (SD)	B [95% CI]	P value
L nucleus accumbens	18389.17 (4198.67)	17974.38 (4067.03)	-311.28 [-802.51,179.94]	1.00
L amygdala	27749.78 (5904.56)	26374.12 (4220.97)	-701.5 [-1323.83,-79.18]	0.94
L bank of the superior temporal sulcu s	27906.4 (5144.26)	28991.09 (5486.11)	319.03 [-498.78,1136.84]	1.00
L caudal anterior cingulate	99364.08 (15726.24)	97915.09 (13662.45)	553.4 [-106.99,1213.79]	1.00
L caudate	50326.11 (10737.9)	45392.74 (9210.79)	-839.54 [-2637.6,958.52]	1.00
L Cerebellum	64414.45 (12615.79)	65527.85 (9412.9)	-2395.72 [-3623.23,-1168.22]	0.06
L caudal middle frontal gyrus	42232.52 (8374.17)	43018.3 (7785.65)	361.43 [-959.77,1682.64]	1.00
L cuneus	21899.06 (5752.99)	20386.96 (5530.44)	373.33 [-624.59,1371.25]	1.00
L entorhinal	7774.54 (2825.44)	8305.6 (3155.26)	-692.35 [-1382.38,-2.31]	0.99
L frontal pole	63654.04 (9810.32)	65686.99 (9113.06)	328.44 [-28.31,685.19]	1.00
L fusiform	80666.09 (10483.03)	75969.65 (8917.33)	1039.19 [-127.7,2206.07]	1.00
L hippocampus	39019.78 (4848.8)	39767.21 (5136.66)	-2355.24 [-3541.32,-1169.16]	**0.03**
L isthmus cingulate cortex	102919.25 (13855.57)	103540.69 (13199.9)	399.36 [-221.75,1020.48]	1.00
L insula	85450.19 (12367.1)	87898.53 (11749.97)	205 [-1458.06,1868.07]	1.00
L inferior parietal lobule	65169.68 (13043.82)	68417.04 (11012.67)	1232.85 [-253.6,2719.31]	1.00
L inferior temporal gyrus	54732.06 (9355.99)	54006.02 (8059.07)	1594.46 [130.92,3058]	0.98
L lingual	65186.75 (10166.63)	65671.89 (9204.88)	-310.67 [-1374.4,753.05]	1.00
L lateral orbitofrontal	81573.36 (14175.5)	85720.1 (12204.93)	412.34 [-742.74,1567.41]	1.00
L lateral occipital gyrus	55233.55 (11103.85)	53678.09 (10448.94)	2084.49 [465.38,3703.59]	0.64
L medial orbitofrontal	64818.41 (11594.07)	66195.92 (10276.11)	-586.75 [-1858.92,685.41]	1.00
L middle temporal gyrus	53561.93 (9282.26)	52203.42 (9358.82)	781.76 [-550.9,2114.42]	1.00
L pallidum	45527.75 (7555.4)	45552.25 (6237.98)	-637.38 [-1781.45,506.69]	1.00
L paracentral	34559.35 (6502.3)	32038.22 (5001.63)	14.79 [-834.75,864.33]	1.00
L parahippocampal	38140.35 (6238.79)	39152.37 (5686.65)	-1254.28 [-1962.52,-546.04]	0.16
L posterior cingulate cortex	78972.58 (10704.07)	80334.9 (9018.85)	527.69 [-207.9,1263.28]	1.00
L precuneus	57726.65 (12722.2)	57813.28 (12166.32)	687.75 [-522.22,1897.71]	1.00
L pericalcarine	44179.22 (8890.28)	46366.89 (7572.4)	57.89 [-1480.94,1596.72]	1.00
L pars opercularis	21814.5 (4641.77)	23041.06 (4122.26)	1053.46 [40.35,2066.56]	0.99
L pars orbitalis	79455.85 (13276.01)	81279.89 (9722.93)	667.73 [139.83,1195.63]	0.79
L postcentral	120601.68 (17788.59)	123136.37 (10851.74)	897.87 [-508.07,2303.8]	1.00
L precentral	37786.95 (6877.09)	38650.06 (6283.18)	1278.34 [-484.97,3041.66]	1.00
L pars triangularis	131869.5 (15898.46)	134289.9 (17003.15)	504.31 [-296.21,1304.83]	1.00
L putamen	33277.51 (5613.13)	32354.78 (6076.02)	1160.98 [-886,3207.97]	1.00
L rostral anterior cingulate cortex	108012.94 (20363.75)	109460.38 (16610.92)	-390.91 [-1113.38,331.55]	1.00
L rostral middle frontal gyrus	148423.79 (22415.09)	151976.54 (12962.65)	520.86 [-1693.49,2735.2]	1.00
L superior frontal gyrus	74717.74 (12189.97)	75008.9 (9269.08)	1871.64 [-309.67,4052.94]	1.00
L supramarginal gyrus	115783.09 (16917.19)	120692.21 (14209.67)	219.27 [-1085.22,1523.76]	1.00

L superior parietal lobule	65110.31 (9887.31)	66699.57 (7663.55)	2419.54 [502.02,4337.07]	0.75
L superior temporal gyrus	121004.22 (12808.14)	118412.9 (12493.77)	873.03 [-181.7,1927.75]	1.00
L thalamus	13019.88 (3838.87)	13361.03 (3825.2)	-1262.04 [-2824.18,300.1]	1.00
L temporal pole	15261.47 (2640.69)	15371.1 (2645.78)	224.18 [-242.7,691.07]	1.00
L transverse temporal	20868.93 (5265.57)	20675.17 (4720.58)	62.04 [-264.97,389.05]	1.00
R nucleus accumbens	31892.98 (6450.73)	31072.06 (5572.3)	-171.09 [-768.28,426.09]	1.00
R amygdala	32237.61 (7672.41)	32444.17 (7190.9)	-415.16 [-1151.63,321.31]	1.00
R bank of the superior temporal sulc us	30873.63 (6367.49)	31918.59 (5336.83)	8.13 [-906.17,922.43]	1.00
R caudal anterior cingulate	106170.67 (17107.12)	102932.17 (13602.37)	519.6 [-200.64,1239.84]	1.00
R caudate	49935.46 (11374.47)	45116.28 (9144.79)	-1699.2 [-3567.05,168.65]	1.00
R Cerebellum	63324 (11228.14)	64026.49 (9839.43)	-2346.37 [-3607.42,-1085.32]	0.08
R caudal middle frontal gyrus	45187.61 (7423.23)	45031.97 (6544.54)	211.1 [-1080.71,1502.9]	1.00
R cuneus	22180.32 (7083.62)	20729.37 (5786.77)	-84.96 [-946.47,776.55]	1.00
R entorhinal	9829.98 (3582.88)	10536.21 (3641.89)	-691.35 [-1482.87,100.16]	1.00
R frontal pole	65106.13 (10536.38)	67015.19 (9005.85)	416.62 [-17.87,851.11]	1.00
R fusiform	87179.09 (12306.75)	82671.57 (11718.9)	806.36 [-390.03,2002.75]	1.00
R hippocampus	35932.18 (5375.78)	36768.52 (4484.05)	-2305.62 [-3775.03,-836.21]	0.25
R isthmus cingulate cortex	106980.78 (18994.13)	105796.18 (17065.22)	445.55 [-160.34,1051.44]	1.00
R insula	93015.81 (11744.73)	94052.43 (10003.75)	-691.48 [-2901.4,1518.45]	1.00
R inferior parietal lobule	65640.18 (12695.82)	66573.13 (9903.84)	417.29 [-912,1746.58]	1.00
R inferior temporal gyrus	59333.64 (9108.95)	58330.36 (7203.8)	481.94 [-898.41,1862.3]	1.00
R lingual	71098.94 (11251.34)	71108.06 (11217.79)	-470.73 [-1470.24,528.77]	1.00
R lateral orbitofrontal	84288.7 (10590.29)	88984.61 (10395.61)	-3.27 [-1393.76,1387.21]	1.00
R lateral occipital gyrus	48282.44 (8692.18)	48062.14 (8813.03)	2221.27 [932.73,3509.82]	0.13
R medial orbitofrontal	69895.41 (12283.73)	70355.41 (10301.23)	-36.37 [-1094.57,1021.83]	1.00
R middle temporal gyrus	54167.02 (10715.81)	54762.44 (9892.68)	358.48 [-1007.64,1724.6]	1.00
R pallidum	50028.63 (7548.67)	49469.08 (5741.22)	323.55 [-934.7,1581.79]	1.00
R paracentral	35641.03 (6563.57)	33121.44 (5695.5)	-292.12 [-1111.14,526.9]	1.00
R parahippocampal	38764.94 (6305.75)	39503.5 (5790)	-1244.24 [-1995.59,-492.88]	0.14
R posterior cingulate cortex	81153.17 (9862.27)	82135.35 (8854.76)	399.16 [-345.15,1143.48]	1.00
R precuneus	61830.88 (10683.31)	61520.67 (11118.76)	320.28 [-819.58,1460.14]	1.00
R pericalcarine	42922.5 (7286.31)	43774.25 (7472.63)	-227.16 [-1579.33,1125.02]	1.00
R pars opercularis	25948.65 (5260.2)	26237.13 (4826.96)	293.41 [-607.08,1193.91]	1.00
R pars orbitalis	80831.79 (12135.72)	81440.85 (9402.37)	165.26 [-454.64,785.15]	1.00
R postcentral	121139.3 (15534.72)	122700.89 (10633.16)	193.31 [-1118.84,1505.46]	1.00
R precentral	40403.43 (6248.98)	40997.79 (7602.87)	644.69 [-966.07,2255.45]	1.00
R pars triangularis	135916.46 (17490.07)	140070.36 (19319.68)	316.19 [-552.1,1184.49]	1.00
R putamen	27236.12 (5049.53)	27443.49 (4655.53)	1835.75 [-431.77,4103.28]	1.00
R rostral anterior cingulate cortex	113566.14 (20442.61)	113761.1 (17211.38)	131.27 [-468.1,730.65]	1.00
R rostral middle frontal gyrus	144200.13 (19088.73)	147624.88 (13601.67)	-109.14 [-2397.05,2178.78]	1.00
R superior frontal gyrus	71723.12 (10958.64)	72446.73 (9737.84)	1716.21 [-289.11,3721.53]	1.00
R supramarginal gyrus	113413.8 (15354.8)	118268.35 (13268.01)	331.24 [-931.3,1593.79]	1.00
R superior parietal lobule	60595.97 (8707.41)	61248.33 (9761.85)	2233.3 [475.8,3990.81]	0.72
R superior temporal gyrus	113115.18 (12392.05)	108095.55 (10519.94)	429.43 [-695.35,1554.2]	1.00

R thalamus	14454.45 (5107.53)	14485.12 (3962.85)	-2416.64 [-3817.04,-1016.23]	0.09
R temporal pole	13632.88 (2488.46)	13309.64 (2202.04)	47.6 [-506.26,601.46]	1.00
R transverse temporal	18389.17 (4198.67)	17974.38 (4067.03)	-133.81 [-416.27,148.65]	1.00
R: right, L:left. All models adjusted for age, motion, study. [a] P values permutation-corrected for multiple comparisons.				

Table S5: Outcomes of between-group analyses of node efficiency

	Efficiency			
Node	HC	AN	Effect of Diagnosis	
	Mean (SD)	Mean (SD)	B [95% CI]	P value
L nucleus accumbens	108.34 (23.8)	107.93 (26.45)	-0.74 [-3.81,2.33]	1.00
L amygdala	137.77 (24.68)	133.7 (21.52)	-1.97 [-4.82,0.87]	1.00
L bank of the superior temporal sulcus	132.92 (28.63)	139.28 (27.8)	3.13 [-0.36,6.62]	1.00
L caudal anterior cingulate	542.95 (92.37)	536.52 (77.5)	3.71 [-0.27,7.7]	1.00
L caudate	185.58 (38.96)	178.83 (43.05)	-3.75 [-14.18,6.69]	1.00
L cerebellum	336.19 (61.18)	334.19 (44.93)	-2.91 [-7.98,2.16]	1.00
L caudal middle frontal gyrus	120.53 (27.55)	122.65 (27.49)	-0.77 [-7.26,5.72]	1.00
L cuneus	68.47 (20.03)	61.98 (16.93)	1.06 [-2.35,4.47]	1.00
L entorhinal	38.34 (13.44)	40.74 (15.63)	-3.09 [-5.33,-0.85]	0.56
L frontal pole	253.23 (44.84)	259.69 (45.57)	1.54 [-0.2,3.28]	1.00
L fusiform	378.63 (46.09)	360.85 (50.6)	3.31 [-2.29,8.91]	1.00
L hippocampus	303.26 (45.99)	307.04 (46.05)	-8.4 [-14.39,-2.41]	0.56
L isthmus cingulate cortex	608 (81.69)	617.23 (78.25)	2.15 [-3.53,7.84]	1.00
L insula	433.4 (69.49)	444.78 (59.42)	4.14 [-5.7,13.97]	1.00
L inferior parietal lobule	311.74 (59.87)	326.72 (51.97)	6.26 [-1.58,14.1]	1.00
L inferior temporal gyrus	188.94 (31.39)	185.15 (30.89)	7.58 [0.72,14.44]	0.96
L lingual	265.95 (43.05)	279.31 (38.37)	-1.53 [-5.35,2.28]	1.00
L lateral orbitofrontal	312.51 (55.58)	318.59 (57.49)	7.6 [2.79,12.42]	0.23
L lateral occipital gyrus	291.95 (77.28)	285 (77.7)	3.44 [-3.49,10.37]	1.00
L medial orbitofrontal	264.73 (48.53)	272.43 (50.83)	-2.04 [-11.17,7.09]	1.00
L middle temporal gyrus	411.84 (69.49)	409.97 (69.73)	5.05 [-0.95,11.05]	1.00
L pallidum	235.42 (40.27)	236.15 (32.11)	0.1 [-8.46,8.65]	1.00
L paracentral	146.45 (35.36)	137.82 (32.21)	0.72 [-3.68,5.12]	1.00
L parahippocampal	276.47 (51.42)	279.08 (42.94)	-3.95 [-8.1,0.2]	1.00
L posterior cingulate cortex	493.3 (71.52)	494.29 (60.35)	1.63 [-4.16,7.41]	1.00
L precuneus	146.91 (31.55)	146.88 (34.06)	1.21 [-6.78,9.2]	1.00
L pericalcarine	247.61 (50.83)	259.63 (38.94)	0.22 [-3.87,4.3]	1.00
L pars opercularis	85.83 (20.97)	93.82 (18.94)	6.75 [1.3,12.21]	0.82
L pars orbitalis	319.27 (56.3)	329.63 (43.71)	4.35 [1.96,6.73]	0.04
L postcentral	544.52 (88.12)	550.62 (60.61)	5.84 [-0.26,11.94]	1.00
L precentral	176.92 (35.63)	184.24 (28.96)	4.13 [-4.92,13.18]	1.00
L pars triangularis	758.28 (90.37)	780.56 (89.33)	4.4 [0.57,8.22]	0.91
L putamen	193.09 (45.6)	188.81 (41.57)	12.02 [1,23.04]	0.96
L rostral anterior cingulate cortex	496.01 (80.16)	508.25 (62.38)	-1.64 [-6.97,3.69]	1.00
L rostral middle frontal gyrus	744.56 (110.72)	757.29 (79.5)	6.68 [-1.93,15.29]	1.00
L superior frontal gyrus	369.12 (62.46)	369.43 (52.46)	7.92 [-3.65,19.49]	1.00
L supramarginal gyrus	591.91 (87.75)	612.48 (71.92)	0.87 [-6.1,7.84]	1.00
L superior parietal lobule	317.55 (50.69)	324.96 (42.85)	11.28 [1.59,20.97]	0.90
L superior temporal gyrus	904.32 (94.06)	896.91 (91.78)	4.39 [-1.23,10.01]	1.00
L thalamus	45.66 (13.96)	46.28 (12.86)	-3.21 [-14.67,8.24]	1.00

L temporal pole	84.49 (17.81)	84.8 (14.96)	0.47 [-1.14,2.09]	1.00
L transverse temporal	127 (29.79)	124.47 (34.72)	0.16 [-1.84,2.17]	1.00
R nucleus accumbens	158.63 (26.36)	153.92 (28.57)	-1.54 [-5.53,2.44]	1.00
R amygdala	145.4 (31.33)	152.3 (31)	-2.29 [-5.69,1.1]	1.00
R bank of the superior temporal sulcus	204.01 (37.61)	211 (33.76)	3.12 [-0.73,6.98]	1.00
R caudal anterior cingulate	592.16 (95.06)	576.39 (83.85)	3.76 [-0.63,8.15]	1.00
R caudate	181.98 (44.44)	175 (39.41)	-7.99 [-18.94,2.96]	1.00
R cerebellum	329.08 (51.75)	331.07 (45.95)	-3.05 [-8.18,2.08]	1.00
R caudal middle frontal gyrus	125.22 (23.34)	123.98 (23.35)	0.91 [-5.09,6.91]	1.00
R cuneus	64.31 (21.71)	60.74 (19.53)	-0.49 [-3.37,2.38]	1.00
R entorhinal	43.85 (14.49)	47.83 (17.66)	-1.57 [-4.06,0.92]	1.00
R frontal pole	238.31 (41.92)	244.59 (43.45)	2.39 [0.45,4.33]	0.81
R fusiform	437.99 (54.61)	422.71 (58.46)	2.47 [-2.81,7.75]	1.00
R hippocampus	266.9 (47.25)	272.49 (39.67)	-7.32 [-14.31,-0.33]	0.99
R isthmus cingulate cortex	633.25 (102.03)	616.3 (90.71)	3.25 [-2.05,8.56]	1.00
R insula	485.51 (66.35)	492.77 (64.71)	-8.5 [-20.27,3.27]	1.00
R inferior parietal lobule	290.53 (43.91)	293.98 (47.75)	3.91 [-4.13,11.95]	1.00
R inferior temporal gyrus	203.41 (33.69)	197.77 (32.39)	2.2 [-3.46,7.86]	1.00
R lingual	310.03 (66.11)	325.07 (65.58)	-2.61 [-6.68,1.46]	1.00
R lateral orbitofrontal	314.99 (49.67)	326.18 (50.65)	7.75 [-0.39,15.9]	1.00
R lateral occipital gyrus	220.09 (63.02)	219.91 (55.8)	5.87 [-0.27,12.02]	1.00
R medial orbitofrontal	285.7 (48.4)	291.69 (51.92)	0.2 [-7.03,7.43]	1.00
R middle temporal gyrus	408.27 (70.74)	415.49 (65.06)	4.44 [-1.53,10.41]	1.00
R pallidum	265.02 (37.64)	264.2 (37.23)	4.26 [-4.05,12.57]	1.00
R paracentral	159.26 (37.48)	147.25 (35.91)	0.07 [-4.53,4.67]	1.00
R parahippocampal	278.15 (49.32)	282.27 (42.84)	-5.69 [-10.19,-1.19]	0.78
R posterior cingulate cortex	491.76 (61.46)	492.25 (50.21)	2.32 [-3.32,7.96]	1.00
R precuneus	147.23 (26.73)	146.21 (29.01)	0.44 [-6.39,7.27]	1.00
R pericalcarine	229.53 (41.75)	234.66 (37.31)	-0.49 [-3.93,2.96]	1.00
R pars opercularis	99.61 (22.91)	104.68 (24.95)	2.58 [-2.29,7.45]	1.00
R pars orbitalis	328.2 (47)	325.74 (44.65)	2.96 [0.04,5.88]	0.99
R postcentral	546.07 (70.51)	547.96 (59.81)	-0.81 [-6.43,4.81]	1.00
R precentral	189.55 (36.31)	195.84 (36.66)	1.6 [-6.36,9.57]	1.00
R pars triangularis	780.61 (97.23)	807.56 (97.44)	3.72 [-0.73,8.17]	1.00
R putamen	147.41 (23.61)	149.63 (24.63)	13.5 [1.43,25.57]	0.96
R rostral anterior cingulate cortex	519.56 (76.61)	529.03 (70.03)	1.33 [-1.64,4.29]	1.00
R rostral middle frontal gyrus	732.98 (98.9)	747.13 (80.65)	5.32 [-3.62,14.25]	1.00
R superior frontal gyrus	357.59 (53.96)	354.35 (54.5)	8.25 [-2.7,19.21]	1.00
R supramarginal gyrus	593.27 (78.13)	609 (65.76)	-1.19 [-7.85,5.47]	1.00
R superior parietal lobule	301.57 (51.46)	306.01 (46.36)	8.38 [-0.32,17.07]	1.00
R superior temporal gyrus	867.27 (91)	840.48 (83.83)	2.83 [-3.07,8.73]	1.00
R thalamus	45.89 (12.88)	46.42 (12.41)	-12.91 [-23.63,-2.19]	0.89
R temporal pole	78.71 (18.14)	79.71 (16.32)	0.43 [-1.11,1.98]	1.00
R transverse temporal	108.34 (23.8)	107.93 (26.45)	0.65 [-1.44,2.74]	1.00

R: right, L:left. All models adjusted for age, motion, study. [a] P values permutation-corrected for multiple comparisons

Table S6: Node metrics by diagnostic group and study

	Study 1		Study 2		Study 3		Study 4		Study 5	
STRENGTH	HC (N=20)	AN (N=23)	HC (N=25)	AN (N=26)	HC (N=13)	AN (N=16)	HC (N=35)	AN (N=30)	HC (N=26)	AN (N=52)
	Mean (SD)	Mean (SD)	Mean (SD)	Mean (SD)	Mean (SD)	Mean (SD)	Mean (SD)	Mean (SD)	Mean (SD)	Mean (SD)
L nucleus accumbens	18120.66 (4985.79)	18241.2 (3427.16)	18607.14 (3370.19)	17480.14 (4277.6)	17138.18 (4739.15)	17455.58 (4741.11)	17583.02 (3164.91)	16668.47 (3766.61)	20096.82 (4914)	19016.51 (4035.56)
L amygdala	27110.76 (4043.22)	27119.45 (4652.87)	27858.4 (5870.55)	25733.41 (4092.42)	29984.98 (5355.73)	26274.17 (4930.81)	26450.65 (5607.27)	26915.77 (4606.87)	28768.11 (7492.79)	26083.08 (3676.26)
L bank of the superior temp oral sulcus	30531.89 (6242.96)	31583.26 (7234.34)	32071.4 (9448.37)	30439.31 (4786.48)	29954.7 (7630.89)	33211.54 (4565.14)	30198.17 (6353.54)	32322.76 (5603.19)	31870.14 (6004.48)	31687.99 (7091.65)
L caudal anterior cingulate	27306.84 (7413.89)	29419.15 (4970.6)	27998.58 (4504.62)	28805.8 (4879.98)	27422.43 (3252.56)	30349.28 (5328.27)	27731.77 (5215.98)	29618.5 (6005.16)	28756.04 (4512.54)	28114.54 (5768.88)
L caudate	98916.97 (13947.24)	100760.67 (12562.6)	98986.55 (14751.6)	99580.39 (18610.71)	94802.77 (20383.3)	100392.73 (11271.09)	96425.4 (13562.3)	95962.12 (11834.76)	106307.57 (16960.1)	96188.18 (12931.44)
L Cerebellum	49203.12 (10453.79)	45719.77 (9340.36)	50812.53 (11418.39)	47762.12 (9278.44)	51523.54 (15751.79)	44918.23 (8315.8)	51152.35 (9735.77)	43835.94 (8210.36)	49011.29 (9124.67)	45107.56 (9993.86)
L caudal middle frontal gyru s	61435.17 (15865.95)	68056.38 (4557.02)	67179.27 (14257.95)	61041.31 (8863.67)	63481.96 (19018.48)	68597.31 (7432.49)	66596.96 (7630.98)	61726.51 (10133.59)	61575.99 (9038.66)	67901.35 (10059.43)
L cuneus	43061.31 (6805.1)	43813.26 (9743.69)	43011.29 (8442.26)	41803.14 (7420.29)	41019.24 (12460.97)	44123.91 (4415.42)	42634.64 (8668.02)	41456.17 (8625.88)	40911.49 (6834)	43835.3 (7348.74)
L entorhinal	22609.84 (6329.62)	19816.93 (6004.43)	22021.78 (5225.33)	20343.35 (5976.53)	23363.73 (5639.95)	20263.33 (7034.06)	21708.47 (5814.26)	22358.29 (4625.79)	20758.57 (5947.67)	19561.63 (4964.37)
L frontal pole	8246.91 (2662.85)	7651.07 (2551.95)	6861.36 (2938.93)	8737.33 (2328.09)	7654.15 (2977.57)	10336.71 (3974.95)	8588.87 (2642.63)	9034.91 (2857.16)	7253.21 (2831.14)	7333.52 (3299.46)
L fusiform	64703.85 (14395.52)	63592.1 (10392.25)	64161.01 (10689.48)	66947.98 (9029.14)	62336.1 (9489.62)	68055.27 (8904.78)	63284.19 (6658.41)	65994.77 (8028.14)	63515.85 (9016.67)	65076.82 (9286.47)
L hippocampus	82284.38 (10817.37)	75211.38 (9029.25)	80021.31 (10038.54)	76582.73 (9296.58)	82191.26 (11711.06)	74679.83 (6415.19)	77496.36 (11562.73)	77522.28 (9356.27)	83545.61 (7678.36)	75499.62 (9252.53)
L isthmus cingulate cortex	40075.55 (6911.84)	39429.08 (4710.28)	37933.48 (5029.91)	40970.89 (4336.9)	38623.9 (2459.82)	40026.16 (5368.09)	39034.66 (3591.14)	39827.74 (6695.26)	39430.07 (5253.9)	39200.32 (4651.64)
L insula	103575.5 (9785.36)	103711.6 (14635.41)	102624 (17580.34)	100756.2 (11552.27)	103934.6 (7598.45)	107063.8 (13630.9)	100282.8 (9884.63)	105779.8 (14453.28)	105739.7 (18896.72)	102481.5 (12473.77)
L inferior parietal lobule	85958.8 (11021.1)	86302.28 (7293.14)	86130.68 (11427.54)	87346.52 (13076.33)	84582.68 (23653.73)	91542.85 (7212.73)	87442.77 (7918.73)	86018.96 (10649.47)	82156.05 (11509.91)	88843.61 (14150.01)
L inferior temporal gyrus	64669.01 (16685.46)	68228.13 (16276.89)	65047.99 (12310.55)	67738.11 (8088.38)	63932.45 (17760.48)	71184.53 (10768.38)	67905.35 (10962.78)	67516.28 (10763.1)	62607.82 (10621.66)	68508.18 (9885.21)
L lingual	56163.35 (10290.29)	52756.15 (7544.96)	53753.33 (9384.62)	56496.7 (8709.53)	54672.75 (11147.62)	56413.83 (4854.13)	54738.79 (8812.12)	53993.35 (8469.32)	54592.74 (8958.88)	52579.94 (8289.36)
L lateral orbitofrontal	64803.14 (11285.9)	66031.64 (8550.86)	66167.7 (9319.79)	63280.91 (7806.77)	61085.29 (14410.24)	73404.52 (9030)	66746.4 (9086.65)	68270.9 (8408.95)	64489.8 (9053.79)	62829.58 (9111.05)
L lateral occipital gyrus	83405.39 (18063.29)	86630.25 (11695.85)	83818.6 (9147.94)	84558.88 (11930.55)	80170.27 (23079.42)	91018.84 (8006.8)	82214.8 (10010.83)	82750.4 (13464.19)	77843.31 (14296.17)	85981.06 (12672.33)
L medial orbitofrontal	55463.02 (10440.02)	53384.87 (11211.71)	53511.45 (12217.3)	53192.8 (9264.37)	54229.72 (8158.08)	58990.1 (8316.64)	57102.36 (12104.75)	56791.64 (11182.52)	54699.09 (10765.18)	50619.68 (10027.84)
L middle temporal gyrus	65222.15 (15220.49)	63606.16 (8334.49)	63624.46 (9399.08)	67447.68 (10525.25)	65114.49 (16785.54)	71410.92 (6766.21)	67050.32 (9063.45)	66280.68 (10504.75)	62503.36 (10606.17)	65061.99 (11320.27)
L pallidum	55291.06 (8017)	52212.66 (8177.98)	51165.26 (10251.98)	55424.29 (9574.5)	53673.85 (10964.71)	50371.43 (6983.36)	50994.23 (7484.76)	53468.04 (11594)	57936.89 (9260.4)	50422.99 (8698.33)
L paracentral	45794.94 (11134.63)	46674.54 (4013.46)	45195.18 (6348.97)	44743.26 (5146.24)	44096.08 (12375.02)	46679.33 (4282.45)	44897.87 (5390.46)	46476.73 (7130.36)	47205.75 (4332.71)	44580.21 (7375.29)

STRENGTH	Study 1 HC (N=20) Mean (SD)	Study 1 AN (N=23) Mean (SD)	Study 2 HC (N=25) Mean (SD)	Study 2 AN (N=26) Mean (SD)	Study 3 HC (N=13) Mean (SD)	Study 3 AN (N=16) Mean (SD)	Study 4 HC (N=35) Mean (SD)	Study 4 AN (N=30) Mean (SD)	Study 5 HC (N=26) Mean (SD)	Study 5 AN (N=52) Mean (SD)
L parahippocampal	34906.31 (5535.98)	32148.58 (5498.47)	34546.74 (7133.15)	32597.78 (3875.34)	35068.43 (7671.86)	31095.23 (4214.3)	33631.75 (5082.4)	32389.74 (5644.11)	35298.74 (7876.36)	31796.98 (5224.72)
L posterior cingulate cortex	37780.12 (10109.98)	39975.02 (4200.59)	37704.22 (5290.53)	38687.02 (5148.39)	37301.29 (3103.35)	40534.65 (6038.28)	38622.31 (5589.51)	39353.99 (6274.6)	38607.52 (5553.19)	38479.56 (6112.22)
L precuneus	80889.21 (15190.91)	81638.47 (9243.29)	77408.17 (8189.93)	81425.33 (8226.08)	77947.77 (18561.01)	82327.13 (8522.44)	78181.18 (6975.36)	80330.78 (8883.8)	80580.26 (7745.78)	78602.48 (9534.73)
L pericalcarine	58009.14 (11091.17)	59841.53 (14133.56)	59350.63 (14283.83)	56158.21 (11532.32)	55178.1 (17022.86)	61362.34 (8433.75)	57842.78 (12347.2)	56377.72 (12417.13)	57065.77 (11027.84)	57479.9 (12460.16)
L pars opercularis	42610.18 (10030.15)	47875.39 (6727.17)	44242.49 (7698.35)	45525.6 (6902.9)	43336.4 (14854.57)	49912.86 (7488.49)	45768.18 (5754.96)	43295.73 (9042.63)	43607.78 (9084.38)	46801.06 (6871.1)
L pars orbitalis	21651.21 (4433.45)	23062.06 (3365.92)	22630.46 (4054.76)	21750.08 (4079.41)	20221.6 (6005.62)	25844.96 (3401.58)	23245.13 (4771.19)	23107.07 (3735.39)	20026.12 (3808.63)	22776.43 (4568.83)
L postcentral	77606.97 (18282.07)	82563.76 (5401.55)	75698.95 (10472.06)	82932.74 (10766.22)	80177.62 (24161.76)	86450.31 (9239.2)	81904.99 (7617.76)	79323.75 (11084.14)	80832.67 (9272.88)	79423.25 (9485.21)
L precentral	119038.7 (25888.21)	124329.7 (7463.27)	119034.4 (16356.2)	122415.6 (14127.26)	117262 (32461.51)	130576.6 (8528.62)	123284.5 (9589.49)	122736.9 (9114.98)	121369.3 (8696.49)	120910.1 (11099.51)
L pars triangularis	37401.83 (6040.36)	38433.56 (6703.55)	36976.48 (6140.67)	38887.72 (5673.67)	35185.34 (10185.46)	43359.74 (4715.1)	40727.5 (5957.47)	37159.48 (5168.33)	36204.87 (6498.56)	38037.8 (6886.68)
L putamen	131347 (10276.41)	136297.5 (15643.36)	132219.1 (14418.1)	132607 (16543.39)	129723.2 (28256.69)	137269.3 (16597.15)	129961.7 (14315.29)	134501.2 (18090.64)	135576.5 (15137.05)	133204.7 (17706.8)
L rostral anterior cingulate c ortex	32979.55 (6252.29)	32175.93 (5755.79)	33694.78 (6770.98)	31737.14 (5847.3)	32365.01 (3365.58)	34248.75 (4945.73)	32634.8 (4430.58)	34347.55 (8127.06)	34426.92 (6342.57)	31010.28 (4936.68)
L rostral middle frontal gyru s	107680.2 (17841.41)	111583.3 (16481.68)	114109.3 (25954.95)	100323.5 (8323.11)	102127.8 (29041.76)	121793.7 (8758.29)	111164.2 (15706.39)	105607.4 (14597.51)	101107.4 (14476.3)	111517.9 (19740)
L superior frontal gyrus	144698.6 (33193.34)	155218.7 (10527.35)	151823.3 (18186.76)	147498.2 (12302.45)	143899.3 (37061.29)	160776.8 (12150.05)	153050 (13816.22)	151010.8 (15974.6)	144055.2 (14648.74)	150631.1 (11351.13)
L supramarginal gyrus	73696.96 (15142.79)	75395.64 (5803.64)	74031.23 (8970.66)	74292.69 (9216.36)	73926.15 (21614.89)	79707.83 (8625.42)	76511.21 (9607.13)	75676.62 (10335.42)	74144.59 (9674.12)	73364.9 (9843.3)
L superior parietal lobule	115542.1 (21111.58)	121436.4 (14524.11)	113587.8 (13159.16)	123308.2 (12760.44)	116579.7 (32735.11)	120242.4 (11769.42)	116928 (10453.3)	118397.6 (14340.17)	116139.8 (13255.97)	120517.3 (15575.93)
L superior temporal gyrus	65662.4 (11905.42)	64805.71 (7554.12)	64173.04 (8911.49)	66455.78 (6989.34)	64979.03 (15948.42)	71858.93 (9425.46)	67024.05 (8973.11)	67167.06 (8424.89)	63076.32 (5942.18)	65801.92 (6490.58)
L thalamus	121032.4 (9926.18)	121223.7 (9951.05)	118099.3 (13757.85)	120513.4 (11151.58)	120388.8 (11292.24)	118276.3 (10115.58)	120411.4 (12305.39)	118239.7 (16070.86)	124881.4 (15027.16)	116261.3 (12476.79)
L temporal pole	14110.13 (5073.2)	12611.68 (3501.27)	12994.99 (3385.77)	13174.09 (3159.53)	12049.29 (3907.03)	14937.56 (5960.47)	13652.63 (3141.94)	14102.85 (3706.3)	11838.67 (3845.42)	12872.89 (3439.32)
L transverse temporal	15289.47 (3110.07)	15111.44 (2299.12)	15143.82 (2658.57)	15351.22 (1903.55)	14897.97 (4220.61)	15706.56 (2004.77)	15712.74 (2181.42)	15140.29 (2595.01)	14927.33 (1808.48)	15525.83 (3293.97)
R nucleus accumbens	20316.21 (4999.94)	20815.38 (4935.87)	21073.8 (4024.17)	20638.11 (5302.43)	21830.7 (7871.28)	18653.29 (3757.03)	20039.51 (5006.94)	19343.91 (3629.56)	21732.74 (5475.57)	22021.83 (4865.72)
R amygdala	32073.07 (6178.5)	32806.97 (5615.45)	32988.45 (7412.83)	28720.42 (3949.93)	33874.16 (10849)	31125.5 (5136.87)	29442.08 (4389.91)	32726.1 (5235.12)	33009.78 (4354.83)	30509.83 (6179.38)
R bank of the superior temp oral sulcus	31337.98 (3896.39)	33423.32 (6160.59)	32837.3 (11614.36)	29874.5 (5155.72)	33339.58 (7043.91)	33128.08 (6586.17)	31308.88 (6586.39)	32571.52 (9527.46)	33052.22 (7103.61)	33012.01 (7068.97)
R caudal anterior cingulate	30497.16 (7606.61)	32353.96 (4190.1)	30817.34 (7022.74)	32359.05 (5178.8)	32273.43 (7225.84)	30128.54 (6071.58)	30675.06 (5540.31)	31512.08 (5585.36)	30784.74 (5660.87)	32291.1 (5550.28)
R caudate	104916.31 (12839.01)	107447.96 (11762.12)	104936.15 (17706.04)	105556.14 (17612.72)	106641.28 (26235.85)	99338.74 (7412.13)	101933.82 (14325.64)	101958.41 (13026.03)	113790.74 (16036.44)	101290.27 (13609.9)
R Cerebellum	48208.22 (11243.12)	46186.87 (9765.26)	50447.21 (12227.87)	47959.91 (9760.5)	51358.76 (17004.7)	43317.25 (7337.25)	51157 (9836.28)	42821.55 (8325.83)	48416 (9616.91)	45098.37 (9357.59)

STRENGTH	Study 1 HC (N=20) Mean (SD)	Study 1 AN (N=23) Mean (SD)	Study 2 HC (N=25) Mean (SD)	Study 2 AN (N=26) Mean (SD)	Study 3 HC (N=13) Mean (SD)	Study 3 AN (N=16) Mean (SD)	Study 4 HC (N=35) Mean (SD)	Study 4 AN (N=30) Mean (SD)	Study 5 HC (N=26) Mean (SD)	Study 5 AN (N=52) Mean (SD)
R caudal middle frontal gyrus	62514.5 (15244.21)	66330.21 (5813.12)	65534.47 (11369.96)	60013.88 (8704.51)	64165.6 (13876.9)	65586.74 (10619.93)	64316.75 (8543.72)	59563.27 (8466.6)	60064.07 (9188.09)	67108.69 (10896.52)
R cuneus	44644.13 (5655.46)	45816.2 (5328.84)	46240.01 (10580.42)	44179.04 (5488.77)	45033.93 (8007.03)	46037.47 (8629.32)	46006.92 (5981.61)	43726.81 (6418.27)	43567.69 (6622.52)	45555.17 (6929.99)
R entorhinal	23650.03 (8477.26)	20891.42 (5537.83)	22040.49 (6729.44)	20182.85 (4290.69)	25458.13 (9998.38)	19314.6 (5429.26)	20335.76 (5683.38)	22912.31 (4954.62)	22028.39 (5891.78)	20106.87 (6855.04)
R frontal pole	10228.79 (4104.63)	9769.41 (2707.26)	8465.76 (2936.36)	11602.61 (2912.32)	9577.63 (3179.76)	13188.54 (4695.06)	10625.19 (3498.9)	11121.86 (2817.82)	9890.65 (3885.29)	9188.18 (3813.85)
R fusiform	64544.8 (12439.37)	67236.92 (7725.27)	67282.14 (10923.88)	65780.3 (7870.86)	67840.37 (16172.7)	65186.76 (7662.39)	62809.52 (6214.37)	67257.13 (7915.62)	65170.08 (9874.34)	67957.57 (10972.08)
R hippocampus	90171.46 (15841.84)	83929.66 (14824.28)	87338.84 (10560.23)	81307.55 (9358.92)	89590.66 (19382.9)	79911.09 (4612.33)	83464.95 (10052.34)	83193.5 (11765.68)	88517.67 (8242.04)	83345.39 (12874.99)
R isthmus cingulate cortex	36979.77 (6682.58)	36352.66 (2326.56)	34986.54 (4744.19)	37748.26 (3633.51)	37188.14 (8135.77)	37204.22 (5013.91)	35534.55 (4079.89)	36849.6 (6084.34)	35942.92 (4861.45)	36281.75 (4408.77)
R insula	110118.3 (15762.82)	107014.6 (16839.67)	103962.7 (24330.04)	102058.5 (12898.33)	118866 (24513.07)	102287.8 (16417.87)	104474.6 (12309.99)	108275.9 (18373.47)	104900.4 (18641.67)	106774.9 (18502.15)
R inferior parietal lobule	91957.77 (11586.48)	94105.36 (9682.99)	93119.51 (11584.76)	91298.83 (10692.62)	98034.31 (14873.51)	94379.65 (10788.22)	92004.23 (10528.36)	95417.51 (10306.24)	92582.48 (12150.58)	94517.61 (9494.8)
R inferior temporal gyrus	63678.54 (16159.49)	68723.34 (10978.53)	67220.07 (15288.14)	64325.7 (6263.81)	70475.22 (15226.99)	67112.68 (7434.99)	65579.75 (9279.37)	67608.38 (11958.98)	63293.85 (9278.56)	65982.52 (10307.79)
R lingual	61349.4 (8694.72)	56634.56 (6424.17)	59154.12 (10463.46)	60295.68 (7728.79)	62053.42 (13466.09)	57310.12 (4388.04)	58072.36 (8022.54)	58669.71 (6636.98)	58293.65 (6634.73)	58215.92 (8191.01)
R lateral orbitofrontal	69862.28 (10374.55)	74153.19 (9407.53)	73921.57 (9220.4)	68220.57 (9137.55)	66303.16 (11561.36)	77449.9 (10520.16)	69334.82 (10241.02)	71872.04 (13941.12)	74108.83 (13870.09)	68812.84 (10550.21)
R lateral occipital gyrus	84610.56 (9021.44)	89253.56 (13804.2)	88159.6 (8956.39)	84768.9 (6069.17)	86367.34 (15650.4)	90217.86 (7240.55)	83588.46 (11548.08)	87420.33 (10223.52)	80222.42 (7508.28)	91496.5 (10808.39)
R medial orbitofrontal	46958.67 (8767.99)	49115.85 (9895.68)	47732.81 (6922.24)	47162.36 (7484.3)	45958.14 (10177.82)	53035.72 (8353.98)	49830.68 (8469.6)	49791.14 (7986.4)	48907.17 (9830.14)	45518.13 (8878.16)
R middle temporal gyrus	66556.04 (17770.97)	72465.88 (13663.17)	69823.66 (12404.09)	68997.4 (7410.42)	72780.93 (12802.85)	74195.81 (6626.5)	71097.7 (9618.2)	70997.14 (8740.27)	69471.92 (10223.63)	68549.05 (11350.53)
R pallidum	55205.06 (8614.69)	56660.77 (8699.98)	51491.78 (10935.24)	57966.94 (9459.91)	56451.47 (18577.13)	51076.67 (7129.15)	51427.2 (6838.13)	55397.07 (12375.92)	58486.86 (10044.77)	53088.5 (9348.14)
R paracentral	50367.89 (12304.99)	49867.07 (3909.79)	48551.32 (4750.98)	49745.99 (5796.77)	51327.75 (9789.11)	49392.45 (3859.25)	49539.52 (5465.58)	49864.55 (6853.84)	51197.02 (6378.38)	48950 (6302.67)
R parahippocampal	36638.67 (6472.4)	33612.77 (6337.06)	35066.08 (6340.46)	33091.43 (5664.92)	37226.72 (9769.26)	32855.96 (5576.17)	34285.81 (5414.79)	33836.93 (4707.33)	36457.95 (6443.85)	32588.03 (6110.77)
R posterior cingulate cortex	38734.92 (9093.02)	39339.22 (4324.85)	38914.72 (5203.13)	39039.38 (5123.38)	40095.88 (7359.7)	38882.99 (7629.01)	38032.89 (5367.26)	40597.15 (6787.28)	38963.99 (5651.26)	39368.2 (5545.91)
R precuneus	82691.66 (14950.69)	82844.71 (8143.21)	80335.19 (7638.04)	83211.42 (8850.75)	83662.69 (13047.63)	80638.79 (7612.04)	79084.89 (7855.21)	80393.51 (7088.11)	82285.72 (7376.33)	82748.96 (10399.74)
R pericalcarine	62510.96 (10935.25)	61667.31 (10978.7)	62979.61 (11407.35)	61178.92 (11225.04)	62751.87 (13875.2)	61905.31 (10216.92)	60421.95 (10458.98)	60906.07 (10513.61)	61639.32 (8762.23)	61862.91 (12088.84)
R pars opercularis	43322.08 (11677.33)	44751.19 (6035.77)	43005.68 (4970.07)	42354.42 (6914.21)	45112.66 (8094.82)	45276.2 (8839.98)	42058.07 (5719.79)	41275.79 (8271.59)	42603.74 (6596.39)	45031.32 (7167.2)
R pars orbitalis	25472.94 (5944.76)	26501.35 (5015.06)	26298.34 (4612.07)	25143.85 (4524.7)	25737.38 (6104.5)	27595.84 (6563.24)	27156.83 (5383.33)	25580.09 (4312.41)	24457.59 (4619.5)	26627.89 (4576.24)
R postcentral	79245.57 (17826.97)	83463.96 (6674.14)	79073.64 (9594.87)	81413.38 (7285.62)	85385.96 (14446.48)	82318.13 (15446.64)	78918.97 (9170.59)	79620.92 (8878.63)	84040.35 (10907.01)	81339.78 (9423.23)
R precentral	120900.4 (26470.09)	124371.3 (7405.57)	119223.4 (15423.39)	121980.6 (8419.05)	124660.6 (17654.95)	124925.6 (14606.7)	120692.9 (8760.82)	120351.3 (11750.74)	122005.6 (10628.87)	122993.2 (10844.04)

R pars triangularis	40341.68 (6323.82)	41557.84 (7319.26)	39780.45 (5837.27)	40568.3 (8038.63)	42722.85 (10124.97)	41675.83 (8454.49)	41338.6 (5454.61)	39757.89 (7401.62)	38631.37 (4884.81)	41471.52 (7550.08)
R putamen	132270.4 (13080.37)	148974.4 (24381.13)	136997 (17206.72)	135806 (17764.22)	143383.8 (29329.98)	131781.9 (19676.62)	132882.5 (15931.93)	136604.4 (14824.93)	138032.6 (14599.1)	142814.1 (18364.09)
R rostral anterior cingulate cortex	26321.1 (3020.3)	27646.33 (3380.07)	27446.97 (6244.73)	27566.48 (4829.77)	28069.5 (6959.73)	26540.68 (5810.32)	27370.13 (3989.95)	27940.21 (4781.96)	27140.15 (5474.38)	27283.5 (4722.13)
R rostral middle frontal gyrus	112793.6 (19472.04)	117693.1 (18924.88)	120132.2 (27301.42)	105359 (11692.27)	115242.2 (23440.22)	119856.3 (9459)	113049.8 (14371.85)	110653.1 (14209.19)	107703.8 (18519.38)	116140.6 (20477.54)
R superior frontal gyrus	141236.8 (31876.8)	151003.6 (11905.33)	145452.8 (14847)	146633.1 (14461.24)	145480.2 (25132.64)	151320.1 (13315.16)	147668.9 (12334.23)	143923.8 (14465.04)	139965.7 (13452.32)	147624.6 (13298.65)
R supramarginal gyrus	71075.8 (15687.57)	73351.27 (6400.65)	70223.66 (10667.83)	72385.38 (7925.74)	74912.24 (12565.34)	75082.33 (10688.27)	72257.19 (9074.35)	72130.21 (9577.23)	71349.32 (8623.04)	71448.98 (11559.23)
R superior parietal lobule	111811.9 (26217.03)	119517.2 (9160.8)	111083.2 (11531.47)	118089.5 (11594.01)	120502.2 (17919.24)	113379.5 (14470.94)	113894.4 (8673.85)	116886.8 (15430.95)	112695.9 (13110.52)	120106.7 (13859.62)
R superior temporal gyrus	60028.06 (11272.3)	63202.83 (11372.98)	59826.42 (8340.94)	60175.24 (6823.11)	61297.63 (10538.84)	64851.81 (12361.9)	61455.22 (7019.52)	60721.04 (9188)	60265.25 (8481.18)	60115.82 (9663.38)
R thalamus	113711.4 (9833.97)	110136.6 (10304.26)	110215.2 (11065.48)	110303.3 (10242.2)	115707 (20960.67)	106884.7 (8419.93)	111798.6 (11372.04)	109426.6 (11118.28)	115921.5 (11144.51)	105693.5 (10802.8)
R temporal pole	15146.91 (7102.57)	14882.51 (3954.27)	14100.83 (3441.24)	14035.56 (2646.77)	16737.57 (7715.26)	12887.53 (5444.28)	14984.15 (3841.16)	14810.35 (4124.81)	12407.18 (4100.37)	14838.06 (3901.48)
R transverse temporal	13514.33 (2767.58)	13556.71 (2178.87)	13383.98 (2539.32)	13192.02 (2071.72)	13702.36 (2811.76)	13758.5 (2173.5)	14035.48 (2082.31)	13353.35 (2467.76)	13386.72 (2675.71)	13095.86 (2175.24)
EFFICIENCY	**Study 1**		**Study 2**		**Study 3**		**Study 4**		**Study 5**	
	HC (N=20)	AN (N=23)	HC (N=25)	AN (N=26)	HC (N=13)	AN (N=16)	HC (N=35)	AN (N=30)	HC (N=26)	AN (N=52)
	Mean (SD)	Mean (SD)	Mean (SD)	Mean (SD)	Mean (SD)	Mean (SD)	Mean (SD)	Mean (SD)	Mean (SD)	Mean (SD)
L nucleus accumbens	106.64 (25.59)	109.78 (26.28)	107.85 (21.08)	108.05 (29.28)	105.03 (25.8)	105.67 (23.81)	105.24 (23)	98.96 (23.7)	115.96 (25.06)	112.92 (26.89)
L amygdala	131.27 (15.21)	139.02 (21.16)	139.03 (24.87)	129.05 (19.46)	140.66 (19.23)	137.65 (26.77)	135.01 (26.29)	137.73 (20.93)	143.8 (30.01)	130.14 (20.9)
L bank of the superior temporal sulcus	125.78 (23.89)	142.28 (24.81)	134.78 (28.03)	134.21 (13.17)	129.98 (34.33)	145.46 (25.38)	132.43 (31.97)	140.34 (29.63)	138.74 (25.35)	137.97 (33.8)
L caudal anterior cingulate	181.97 (48.76)	192.89 (36.29)	182.61 (26.9)	191.01 (25.66)	182.6 (21.84)	197.18 (37.98)	181.85 (27.67)	192.25 (35.7)	184.73 (32.29)	183.69 (30.2)
L caudate	539.69 (103.31)	546.6 (73.62)	537.69 (77.84)	547.33 (97.17)	515.38 (119.46)	555.66 (63.36)	531.35 (81.49)	526.63 (68.81)	579.91 (91.78)	526.48 (77.3)
L Cerebellum	182.46 (28.6)	176.08 (34.72)	187.35 (46.2)	185.15 (42.64)	171.99 (36.78)	201.72 (46.7)	195.46 (31.66)	174.94 (38.18)	179.79 (46.91)	172.08 (46.69)
L caudal middle frontal gyrus	324.87 (81.86)	351.74 (39.82)	335.29 (52.13)	322.31 (47.27)	325.81 (95)	356.77 (39.57)	354.2 (43.76)	324.86 (37.64)	326.72 (48.2)	330.8 (47.97)
L cuneus	120.7 (18.67)	122.77 (40.82)	124.65 (34.16)	114.58 (19.08)	110.31 (36.79)	134.6 (24.13)	126.83 (25.11)	117.86 (25.24)	113.08 (22.51)	125.73 (25.13)
L entorhinal	70.09 (21.58)	59.53 (16.13)	69.57 (20.97)	60.16 (15.89)	70.22 (21.42)	65.68 (23.6)	68.34 (15.63)	68.57 (16.51)	65.47 (23.49)	59.02 (14.93)
L frontal pole	40.3 (11.22)	37.66 (12.29)	33.39 (15.65)	43.37 (12.11)	39.96 (16.58)	49.07 (22.77)	43.11 (11.25)	43.24 (11.84)	34.38 (11.84)	36.79 (16.76)
L fusiform	245.97 (38.89)	262.1 (52.84)	258.7 (49.58)	261.7 (37.47)	241.62 (42.55)	270.23 (44.71)	256.35 (39)	256.04 (49.23)	255.16 (53.71)	256.49 (45.03)
L hippocampus	382.73 (40.03)	356.48 (44.62)	372.46 (48.02)	366.94 (55.44)	365.63 (35.8)	373.86 (37.09)	386.23 (51.36)	356.74 (54.97)	377.69 (46.61)	358.09 (52.38)
L isthmus cingulate cortex	303.9 (55.18)	312.29 (42.76)	293.67 (52.78)	316.62 (40.46)	303.13 (28.37)	312.14 (41.57)	308.69 (32.04)	304.33 (59.88)	304.76 (55.49)	299.93 (42.52)
L insula	604.52 (75.13)	634.54 (82.88)	608.42 (94.84)	602.9 (82.25)	611.87 (58.85)	632.43 (80.27)	601.92 (64)	619.76 (88.48)	616.51 (105.79)	610.59 (67.39)
L inferior parietal lobule	425.49 (70.31)	442.05 (41.63)	423.23 (58.5)	453 (63.22)	427.67 (117.6)	462.75 (66.62)	450.21 (60.36)	434.78 (58.99)	429.5 (59.82)	442.1 (62.54)
L inferior temporal gyrus	302.83 (57.85)	333.96 (53.14)	306.52 (49.46)	323.87 (43.12)	308.91 (84.41)	338.48 (54.71)	321.18 (55.35)	327.02 (65.85)	312.31 (64.94)	321.16 (46.42)

EFFICIENCY	**Study 1**		**Study 2**		**Study 3**		**Study 4**		**Study 5**	
	HC (N=20)	AN (N=23)	HC (N=25)	AN (N=26)	HC (N=13)		HC (N=20)	AN (N=24)	HC (N=25)	AN (N=26)
	Mean (SD)	Mean (SD)	Mean (SD)	Mean (SD)	Mean (SD)		Mean (SD)	Mean (SD)	Mean (SD)	Mean (SD)
L lingual	185.4 (25.84)	186.99 (32.24)	191.58 (38.49)	190.71 (29.67)	184.8 (33.67)	195.05 (27.54)	192.97 (30.91)	183 (36.76)	185.75 (28.58)	179.75 (27.93)
L lateral orbitofrontal	260.71 (31.69)	280.48 (34.47)	268.64 (42.41)	273.44 (37.4)	263.35 (55.01)	302.57 (42.37)	275.25 (43.41)	289.7 (38.89)	256.19 (44.62)	268.56 (35.48)
L lateral occipital gyrus	316.65 (46.71)	308.08 (40.9)	315.28 (43.69)	313.78 (48.43)	300.94 (97.59)	352.6 (43.4)	321.63 (44.15)	322.77 (72.22)	300.17 (59.17)	312.77 (60)
L medial orbitofrontal	301.25 (68.31)	276.78 (84.48)	267.77 (85.35)	295.76 (77.92)	298.39 (42.81)	307.67 (61.03)	311.81 (84.92)	293.84 (75.68)	278.07 (74.37)	271.18 (79.9)
L middle temporal gyrus	259.67 (51.18)	268.12 (39.92)	255.88 (51.44)	288.47 (37.64)	276 (70.82)	285.05 (57.11)	273.14 (40.23)	281.26 (53.18)	260.18 (41.12)	257.35 (54.58)
L pallidum	414.21 (75.84)	413.47 (61.05)	391.51 (90.55)	432.62 (71.29)	414.29 (84.53)	403.92 (48.92)	412.65 (54.43)	420.53 (83.66)	427.24 (49.25)	392.86 (67.05)
L paracentral	232.38 (57.75)	246.28 (19.36)	233.59 (34.85)	231.62 (29.71)	235.42 (66.93)	248.88 (24.5)	237.96 (28.81)	239.89 (31.22)	236.09 (25.59)	227.87 (37.96)
L parahippocampal	145.94 (36.02)	137.23 (29.25)	145.27 (38.66)	142.14 (32.55)	141.2 (31.35)	143.27 (33.64)	151.03 (33.4)	138.62 (35.38)	144.42 (37.98)	133.78 (31.64)
L posterior cingulate cortex	271.78 (76.81)	293.66 (37.33)	275.83 (55.41)	275.98 (35.69)	275.45 (32.33)	289.09 (37.77)	282.32 (42.86)	273.19 (48.71)	273.32 (44.41)	274.5 (45.95)
L precuneus	486.74 (103.04)	514.52 (53.01)	486.05 (55.95)	493.88 (44.2)	482.7 (111.4)	518.81 (59.29)	506.66 (46.21)	493.34 (76.2)	492.62 (61.75)	478.54 (57.41)
L pericalcarine	141.98 (27.31)	151.17 (40.74)	152.82 (36.26)	140.92 (28.49)	131.38 (43.71)	163.5 (17.9)	153.59 (29.61)	142.99 (41.73)	143.77 (22.51)	145.09 (31.58)
L pars opercularis	241.95 (64.44)	268.08 (28.69)	243.96 (48.82)	259.49 (32.4)	236.71 (68.49)	280.23 (26.63)	262.3 (34.01)	255.46 (53.62)	241.15 (49.82)	252.03 (37.32)
L pars orbitalis	86.96 (20.26)	91.23 (18.41)	85.45 (20.85)	90.97 (17.84)	83.93 (27.77)	102.61 (17.37)	92.81 (17.67)	95.85 (17.36)	76.9 (19.94)	92.52 (20.73)
L postcentral	309.49 (74.96)	339.68 (26.44)	310.78 (58.4)	335.57 (42.7)	311.24 (86.42)	351.16 (29.72)	336.74 (29.5)	322.02 (49.9)	315.48 (45)	319.97 (47.55)
L precentral	539.82 (124.5)	564.12 (43.64)	540.26 (92.04)	546.55 (61.02)	521.09 (137.2)	586.57 (50.6)	563.89 (48.19)	551.75 (70.08)	537.89 (60.44)	534.97 (59.81)
L pars triangularis	174.7 (41.7)	183.25 (23.06)	168.8 (33.77)	187.97 (26.13)	174.6 (48.83)	195.99 (26.38)	191.39 (29.39)	186.33 (34.24)	168.14 (28.45)	177.98 (29.52)
L putamen	746.89 (82.47)	787.47 (63.83)	753.69 (84.86)	778.1 (93.48)	747 (160)	806.6 (85.82)	772.09 (74.39)	787.74 (91.02)	758.53 (78.71)	766.57 (97.26)
L rostral anterior cingulate c ortex	197.31 (59.53)	184.77 (29.91)	191.78 (43.22)	183.13 (41.83)	194.96 (39.07)	201.82 (36.77)	193.03 (34.7)	200.21 (53.65)	190.23 (54.15)	182.84 (38.46)
L rostral middle frontal gyru s	498.45 (104.9)	511.3 (53.13)	491.9 (71.55)	491.16 (58.37)	475.66 (133.15)	562 (72.3)	513.68 (58.35)	511.64 (68.99)	484.46 (55.66)	496.96 (53.01)
L superior frontal gyrus	728.65 (163.91)	770.8 (61.98)	742.01 (98.36)	755.12 (69.95)	733.51 (170.99)	791.89 (80.71)	768.59 (75.12)	764.78 (92.59)	732.43 (72.19)	737.42 (79.69)
L supramarginal gyrus	361.23 (77.02)	376.33 (25.78)	367.23 (52.82)	368.6 (53.8)	361.69 (101.36)	394.72 (45.83)	380.4 (43.1)	371.35 (59.62)	365.56 (59.12)	357.89 (56.33)
L superior parietal lobule	573.07 (110.52)	614.22 (60.06)	579.91 (75.29)	625.13 (63.88)	582.7 (158.86)	626.93 (71.42)	619.3 (53.57)	611.54 (93.59)	585.66 (63.56)	601.49 (67.06)
L superior temporal gyrus	310.13 (38.97)	324.76 (26.22)	310.57 (61.25)	328.19 (33.65)	308.29 (79.25)	354.04 (42.47)	334.01 (42.53)	322.86 (47.74)	312.45 (36.78)	315.69 (47.1)
L thalamus	899.56 (109.61)	911.76 (77.21)	892.8 (100.25)	901 (77.13)	895.29 (77.53)	912.85 (74.04)	911.11 (85.53)	894.54 (128.39)	914.44 (98.9)	884.77 (85.44)
L temporal pole	51.31 (18.44)	44.14 (10.8)	42.64 (14.9)	44.08 (10.53)	43.79 (11.71)	50.6 (15.8)	47.31 (11.06)	51.04 (14.6)	42.94 (12.96)	44.25 (12.08)
L transverse temporal	80.71 (15.72)	84.96 (9.41)	80.69 (17.03)	85.7 (14.74)	81.35 (24.76)	86.9 (10.35)	88.79 (13.7)	83.63 (17.46)	86.84 (20.45)	84.31 (16.99)
R nucleus accumbens	123.88 (24.79)	125.2 (37.71)	126.26 (23.63)	128.03 (41.64)	134.34 (37.9)	115.82 (32.13)	123.25 (33.76)	117.22 (33.17)	131.49 (29.5)	129.21 (31.22)
R amygdala	153.01 (29.47)	165.82 (27.72)	161.76 (29.78)	145.3 (20.22)	165.44 (35.43)	160.66 (28.69)	153.36 (20.68)	155.99 (28.88)	163.64 (21.44)	149.69 (30.85)

EFFICIENCY	Study 1		Study 2		Study 3		Study 4		Study 5	
	HC (N=20)	AN (N=23)	HC (N=25)	AN (N=26)	HC (N=13)	AN (N=16)	HC (N=35)	AN (N=30)	HC (N=26)	AN (N=52)
	Mean (SD)	Mean (SD)	Mean (SD)	Mean (SD)	Mean (SD)	Mean (SD)	Mean (SD)	Mean (SD)	Mean (SD)	Mean (SD)
R bank of the superior temporal sulcus	138.33 (25.73)	159.88 (31.4)	142.22 (36.84)	147.36 (28.94)	150.57 (24.68)	151.53 (23.16)	147.05 (35.27)	146.95 (34.82)	149.11 (27.54)	154.76 (31.72)
R caudal anterior cingulate	206.79 (51.23)	213.71 (32.93)	197.99 (44.25)	214.15 (26.08)	204.5 (28.26)	207.79 (41.1)	207.35 (32.24)	209.96 (39.24)	202.91 (30.77)	209.8 (32.79)
R caudate	583.25 (90.53)	594.94 (74.2)	583.76 (106.22)	591.58 (112.93)	587.26 (115.55)	567.95 (61.6)	577.99 (82.82)	571.24 (81.86)	628.63 (89.66)	566.16 (78.47)
R Cerebellum	179 (33.34)	173.94 (38.71)	182.96 (52.95)	184.5 (39.75)	172.11 (54.17)	185.07 (36.88)	193.33 (39.64)	170.25 (35.19)	172.99 (43.78)	170.35 (42.51)
R caudal middle frontal gyrus	319.65 (79.63)	349.97 (38.05)	341.22 (52.11)	311.49 (43.59)	337.83 (43.2)	344.54 (43.47)	332.1 (41.26)	318.63 (45.16)	316.24 (39.54)	335.53 (47.43)
R cuneus	122.95 (16.39)	123.97 (25.54)	126.9 (29.49)	123.57 (18.22)	119.52 (23.74)	133.99 (29)	131.53 (21.67)	120.17 (24.19)	119.71 (22.63)	123.32 (22.26)
R entorhinal	68.03 (28.26)	58.01 (22.68)	63.88 (17.4)	60.08 (18.79)	67.82 (26.25)	66.38 (22.56)	64.89 (20.36)	65.2 (16.69)	59.31 (19.7)	57.97 (18.87)
R frontal pole	44.55 (11.86)	44.27 (13.93)	37.11 (15.71)	53.81 (14.34)	44.55 (10.33)	56.09 (21.07)	48.77 (14.89)	51.42 (17.28)	42.79 (14.6)	41.81 (17.84)
R fusiform	225.93 (33.35)	254.44 (47.68)	248.01 (49.49)	229.99 (33.14)	245.12 (53.13)	236.69 (31.82)	232.74 (34.79)	241.69 (46.06)	242.57 (42.33)	251.65 (46.41)
R hippocampus	437.81 (60.01)	435.49 (76.12)	439.83 (50.04)	418.93 (41.66)	437.11 (56.87)	421.94 (30.69)	440.6 (61.39)	413.87 (67)	433.31 (47.11)	424.29 (58.94)
R isthmus cingulate cortex	266.53 (57.5)	271.73 (28.51)	259.61 (49.18)	279.38 (32.04)	279.08 (49.72)	283.51 (46.04)	270.02 (38.22)	273.61 (47.9)	263.91 (48.77)	265.35 (40.1)
R insula	646.87 (83.44)	621.32 (73.32)	607.13 (113.76)	609.88 (100.36)	701.31 (112.42)	602.67 (84.01)	632.42 (75.07)	624.67 (103.59)	614.97 (118.74)	616.65 (89.38)
R inferior parietal lobule	474.51 (91.1)	506.08 (56.68)	477.96 (63.94)	485.44 (67.02)	501.52 (65.95)	489.22 (68.4)	485.11 (54.08)	502.43 (71.82)	493.77 (64.2)	486.08 (62.28)
R inferior temporal gyrus	279.42 (36.61)	307.4 (42.3)	286.12 (48.56)	297.83 (38.33)	305.58 (44.62)	298.33 (51.19)	295.04 (40.06)	295.56 (63.21)	289.72 (49.17)	283.86 (42.33)
R lingual	209.81 (24.28)	189.26 (28.1)	202.93 (44.13)	204.96 (28.09)	202.15 (42.45)	203.88 (20.05)	203.12 (33.91)	198.16 (34.25)	199.97 (23.74)	195.84 (37.62)
R lateral orbitofrontal	295.54 (66.73)	343.41 (57.16)	321.98 (68.41)	314.91 (65.15)	288.71 (51.88)	356.82 (75.06)	311.53 (59.69)	321.81 (68.32)	318.34 (77.42)	314.14 (62.14)
R lateral occipital gyrus	302.57 (41.04)	338.55 (46.1)	321.72 (54.28)	310.25 (34.81)	322.7 (44.65)	334.85 (52.4)	324.32 (51.48)	330.36 (64.92)	301.65 (49.68)	323.6 (48.88)
R medial orbitofrontal	211.29 (61.48)	227.07 (66.41)	211.32 (53.45)	212.85 (51.03)	205.84 (31.76)	243.26 (52.22)	234.09 (73.53)	221.17 (46.38)	223.55 (69.03)	212.35 (58.57)
R middle temporal gyrus	277.8 (53.34)	292.29 (37.25)	276.8 (63.88)	309.45 (28.26)	289.24 (52.07)	306.12 (54.4)	298.81 (31.77)	297.39 (57.89)	280.9 (43.62)	274.81 (58.45)
R pallidum	407.31 (66.66)	422.68 (49.32)	385.25 (88.43)	436.47 (65.41)	417.28 (103.53)	407.03 (49.86)	407.82 (49.63)	420.71 (81.8)	427.26 (57.35)	401.42 (62.82)
R paracentral	261.41 (66.71)	273.93 (27.31)	261.27 (30.92)	267.1 (39.44)	269.59 (27.53)	267.61 (20.23)	268.85 (29.24)	265.77 (44.36)	263.98 (28.38)	256.48 (39.14)
R parahippocampal	156.34 (37.7)	154.4 (37.1)	154.41 (33.45)	150.85 (38.19)	169.61 (48.15)	149.69 (44.29)	160.28 (34.67)	149.61 (37.66)	159.62 (40.5)	140.17 (30.22)
R posterior cingulate cortex	279.59 (67.77)	285.1 (38.1)	283.13 (42.17)	280.53 (39.36)	287.14 (54.16)	281.48 (52.29)	268.91 (43.06)	285.62 (46.82)	280.17 (46.47)	280.19 (42.43)
R precuneus	492.14 (96.5)	497.77 (44.1)	487.88 (61.01)	498.53 (45.73)	495.75 (38.18)	510.66 (47.49)	496.02 (50.49)	486.68 (57.84)	487.49 (54.27)	484.21 (50.62)
R pericalcarine	146.41 (23.8)	141.55 (23.14)	149.75 (31)	146.43 (26.18)	140.47 (26.6)	158.01 (22.52)	150.18 (29.97)	146.74 (35.4)	144.83 (20.25)	144.23 (30.39)
R pars opercularis	230.55 (64.08)	238.56 (30.25)	223.25 (41.41)	233.76 (30.75)	236.79 (23.79)	231.86 (24.83)	230.55 (36.12)	233.56 (47.59)	229.78 (36.67)	234.88 (40.59)
R pars orbitalis	98.2 (23.43)	101.63 (29.48)	98.6 (24.59)	104.41 (23.63)	102.13 (23.45)	108.2 (30.7)	108.81 (21.79)	105.38 (21.49)	88.02 (17.47)	104.68 (24.18)
R postcentral	316.7 (72.84)	339.71 (41.69)	322.84 (40.48)	332.37 (37.05)	335.75 (36.17)	323.1 (26.75)	328.77 (35.35)	324.45 (52.37)	337.68 (47.27)	317.79 (48.33)

EFFICIENCY	Study 1		Study 2		Study 3		Study 4		Study 5	
	HC (N=20)	AN (N=23)	HC (N=25)	AN (N=26)	HC (N=13)	AN (N=16)	HC (N=35)	AN (N=30)	HC (N=26)	AN (N=52)
	Mean (SD)	Mean (SD)	Mean (SD)	Mean (SD)	Mean (SD)		Mean (SD)	Mean (SD)	Mean (SD)	Mean (SD)
R precentral	537.19 (122.53)	561.94 (42.16)	542.91 (64.55)	544.06 (45.98)	548.86 (59.82)	562.37 (31.69)	555.35 (45.57)	549.48 (75.98)	542.05 (57.42)	538.42 (67.86)
R pars triangularis	188.31 (43.11)	193.88 (40.18)	182.8 (36.31)	198.8 (29.37)	199.39 (32.3)	191.15 (32.9)	196.93 (36.64)	200.4 (40.59)	182.16 (31.44)	194.05 (38)
R putamen	756.24 (101.09)	837.36 (83.16)	772.21 (103.67)	809.89 (94.28)	802.99 (126.47)	789.57 (103.49)	795.47 (88.57)	788.49 (105.71)	776.25 (84)	809.75 (98.06)
R rostral anterior cingulate c ortex	147.16 (21.48)	147.55 (17.88)	141.58 (23.9)	148.6 (21.19)	153.77 (28.32)	151.01 (29.67)	152.73 (21.71)	150.36 (23.68)	142.85 (24.32)	150.22 (28.23)
R rostral middle frontal gyru s	510.35 (112.53)	541.32 (83.22)	521.31 (67.57)	510.33 (48.58)	527.66 (68.7)	556.25 (59.55)	523.68 (63.8)	539.02 (80.67)	515.39 (75.82)	518.81 (67.12)
R superior frontal gyrus	719.66 (159.31)	762.43 (67.09)	723.07 (100.44)	754.53 (73.94)	730.98 (86.27)	766.54 (82.71)	751.94 (73.78)	751.52 (81.75)	728.22 (73.82)	728.15 (86.92)
R supramarginal gyrus	352.95 (81.93)	360.11 (30.68)	349.13 (45.89)	360.01 (40.83)	370.46 (55.36)	360.65 (48.92)	367.19 (45.94)	353.85 (55.52)	349.92 (43.75)	347.31 (68.76)
R superior parietal lobule	579.03 (140.14)	617.7 (55.19)	565.41 (59.41)	615.76 (63.31)	611.77 (64.7)	606.62 (62.17)	616.88 (48.14)	613.57 (87.6)	589.98 (58.55)	599.86 (58.7)
R superior temporal gyrus	295.67 (49.88)	312.71 (41.46)	297.09 (64.84)	313.29 (33.63)	302.34 (59.65)	312.76 (35.98)	311.19 (45.11)	297.61 (54.2)	297.1 (43.59)	302.16 (51.83)
R thalamus	872.12 (110.88)	846.66 (81.25)	851.43 (83.95)	851.52 (92.07)	867.92 (105.39)	847.07 (66.44)	866.81 (86.71)	838.07 (93.22)	879.05 (83.72)	831.57 (81.83)
R temporal pole	45.84 (12.6)	45.75 (12.02)	45.69 (13.45)	45.9 (10)	51.82 (19.43)	43.52 (12.83)	48.53 (9.16)	47.95 (13.67)	39.6 (11.13)	47 (13.03)
R transverse temporal	75.45 (15.39)	81.14 (15.04)	79.77 (24.58)	77.52 (15.03)	76.5 (16.12)	79.97 (15.96)	80.43 (14.69)	81.12 (19.69)	78.96 (18.95)	79.29 (15.94)
Global Efficiency	2719.05 (106.9 9)	2788.23 (96.9)	2738.62 (118.0 6)	2720.88 (146.8 1)	2723.42 (90.21)	2817.99 (99.72)	2730.81 (111.1 1)	2723.69 (127.8 4)	2751.24 (97.1)	2718.18 (113.1 2)

Unharmonized data analyses

Table S7: Outcomes of network-based statistics analysis with unharmonized data

HC > AN	Connection	T-statistic for between group difference	P-value for component[a]
	R thalamus - L hippocampus	3.15	0.038
	R thalamus - R hippocampus	3.41	
	R thalamus - R amygdala	3.55	
	L parahippocampal gyrus - R parahippocampal gyrus	3.35	
	R thalamus - R parahippocampal gyrus	3.11	
	R hippocampus - R postcentral gyrus	3.26	
	R paracentral gyrus - R postcentral gyrus	3.34	
	L cerebellum - R cerebellum	3.11	
	R parahippocampal gyrus - R cerebellum	3.54	
**AN > HC**	**Connection**	**T-statistic for between group difference**	**P-value for component**
	L lateral orbitofrontal gyrus - L pars opercularis	3.29	0.018
	L pars opercularis - L pars orbitalis	3.58	
	L caudal anterior cingulate – L pars triangularis	3.12	
	L pars orbitalis - L pars triangularis	3.16	
	L precentral gyrus – R putamen	3.57	
	L pars opercularis - R putamen	3.22	
	L precentral gyrus – L superior temporal gyrus	3.31	
	L pars opercularis - R lateral orbitofrontal gyrus	3.28	
	L lateral orbitofrontal gyrus - R rostral middle frontal gyrus	3.98	
	L lateral orbitofrontal gyrus - R rostral anterior cingulate cortex	3.31	
	L superior temporal gyrus - R putamen	3.21	

Models adjusted for age, motion, study, [a] P values Bonferonni-corrected for multiple comparisons, R: right, L:left

Table S8: Outcomes of between-group analyses of node strength calculated with unharmonized data

	Strength			
**Node**	HC Mean (SD)	AN Mean (SD)	Effect of Diagnosis	P value
L nucleus accumbens	18558.06 (4902.75)	18167.33 (4685.57)	-457.35 [-996.49,81.8]	1.00
L amygdala	28002.01 (6811.12)	26609.86 (4800.53)	-702.6 [-1398.9,-6.31]	1.00
L bank of the superior temporal sulcus	30908.79 (7295.44)	32414.24 (7825.42)	512.4 [-388.57,1413.36]	1.00
L caudal anterior cingulate	28203.15 (5362.87)	29193.31 (5476.48)	591.4 [-69.12,1251.92]	1.00
L caudate	99768.97 (16701.06)	98790.67 (14758.58)	-627.99 [-2499.21,1243.23]	1.00
L Cerebellum	50669.56 (12792.06)	45578.9 (10830.9)	-2004.94 [-3316.51,-693.37]	0.28
L caudal middle frontal gyrus	64931.64 (13160.15)	65955.86 (9490.04)	378.13 [-984.15,1740.41]	1.00
L cuneus	42446.29 (8310.31)	43323.61 (7941.82)	427.27 [-562.08,1416.63]	1.00
L entorhinal	22003.79 (5935.08)	20561.67 (5816.47)	-570.34 [-1277.38,136.7]	1.00
L frontal pole	8187.46 (3153.86)	8172.39 (3600.43)	172.32 [-212.35,557]	1.00
L fusiform	63304.45 (10553.34)	66514.15 (10331.44)	1439.88 [216.8,2662.97]	0.91
L hippocampus	80981.42 (10631.44)	76136.86 (9403.45)	-2162.96 [-3378.72,-947.21]	0.12
L isthmus cingulate cortex	39518.14 (5228.62)	39713.02 (5370.17)	270.03 [-364.51,904.57]	1.00
L insula	103638.18 (13549.19)	104301.78 (14712.42)	171.28 [-1540.6,1883.16]	1.00
L inferior parietal lobule	85545.93 (13645.42)	88698.96 (12347.41)	1337.3 [-192.08,2866.69]	1.00
L inferior temporal gyrus	64807.88 (13191.55)	69494.94 (12162.11)	2206.3 [677.47,3735.14]	0.37
L lingual	55029.18 (10215.03)	54486.07 (8781.58)	-372.32 [-1451.68,707.04]	1.00
L lateral orbitofrontal	65347.24 (9963.84)	66294.54 (9375.84)	648.77 [-524.27,1821.81]	1.00
L lateral occipital gyrus	82407.66 (16505.63)	86231.15 (13573.9)	2110.9 [446.59,3775.22]	0.80
L medial orbitofrontal	55110.33 (11289.52)	54617.59 (10412.72)	-320.3 [-1629.27,988.68]	1.00
L middle temporal gyrus	65243.23 (13253.12)	66246.31 (12116.18)	1056.6 [-298.14,2411.34]	1.00
L pallidum	54286.58 (9905.73)	52479.99 (10366.06)	-427.21 [-1592.81,738.39]	1.00
L paracentral	45969.34 (8200.67)	45949.12 (6096.01)	20.91 [-830.18,872]	1.00
L parahippocampal	34691.92 (6659.53)	32025.75 (5348.64)	-1160.16 [-1879.28,-441.04]	0.23
L posterior cingulate cortex	38588.19 (6656.18)	39435.11 (5829.58)	581.81 [-173.95,1337.57]	1.00
L precuneus	79862.89 (13361.86)	81161.49 (10709.95)	565.5 [-770.12,1901.12]	1.00
L pericalcarine	58077.4 (12957.45)	58337.82 (13042.46)	236.91 [-1296.15,1769.98]	1.00
L pars opercularis	44534.35 (10458.24)	46619.78 (9228.77)	1227.15 [70.17,2384.12]	0.98
L pars orbitalis	21792.06 (4531.07)	23168.59 (4338.25)	813.48 [276.11,1350.84]	0.41
L postcentral	80374.29 (16096.74)	81255.62 (10324.34)	721.8 [-807.32,2250.91]	1.00
L precentral	121487.47 (19446.12)	123787.87 (10924.38)	1346.86 [-522.99,3216.7]	1.00
L pars triangularis	37689.02 (6965.84)	38916.93 (6845.45)	778.04 [-58.94,1615.03]	1.00
L putamen	132502.28 (16407.71)	135200.28 (17238.08)	1280.41 [-803.06,3363.88]	1.00
L rostral anterior cingulate cortex	33770.91 (6387.86)	32389.53 (6051.56)	-499.62 [-1241.12,241.87]	1.00
L rostral middle frontal gyrus	107928.87 (20835.95)	111073.15 (18504.95)	961.87 [-1328.74,3252.48]	1.00
L superior frontal gyrus	149162.48 (23496.81)	152823.18 (12476.87)	2145.87 [-65.15,4356.9]	1.00
L supramarginal gyrus	75378.51 (13172.1)	75123.65 (9131.86)	123.61 [-1220.87,1468.1]	1.00
L superior parietal lobule	116685.05 (18257.83)	121345.76 (15272.21)	2145.74 [115.99,4175.49]	0.99
L superior temporal gyrus	65479.37 (11773.62)	66996.22 (9541.44)	1062.91 [-120.37,2246.2]	1.00
L thalamus	121895.45 (14050.93)	119144.73 (13463.65)	-1139.65 [-2724.51,445.21]	1.00
L temporal pole	13354.29 (4086.51)	13371.52 (3998.44)	148.27 [-336.18,632.73]	1.00
L transverse temporal	15251.27 (2763.83)	15591.65 (2898.93)	170.44 [-160.03,500.91]	1.00
R nucleus accumbens	21126.77 (5612)	20859.52 (5117.06)	-232.17 [-859.81,395.47]	1.00
R amygdala	32460.93 (7719.37)	31410.13 (6909.62)	-507.72 [-1326.5,311.05]	1.00

R bank of the superior temporal sulcus	32292.71 (7870.74)	33369.01 (8852.74)	165.48 [-823.96,1154.93]	1.00
R caudal anterior cingulate	31561.87 (6656.2)	31755.1 (5888.15)	337.19 [-408.93,1083.31]	1.00
R caudate	106867.21 (17756.45)	103698.89 (15577.06)	-1421.67 [-3350.92,507.58]	1.00
R Cerebellum	50132.91 (13651.3)	45229.68 (10697.54)	-1862.31 [-3214.21,-510.42]	0.63
R caudal middle frontal gyrus	63493.36 (11891.57)	64826.53 (10603.83)	452.52 [-872.78,1777.83]	1.00
R cuneus	45166.72 (7268.76)	45279.48 (6777.15)	-2.32 [-860.92,856.28]	1.00
R entorhinal	22577.82 (8396.87)	20885.22 (6224.55)	-715.93 [-1575.84,143.98]	1.00
R frontal pole	10049.2 (3763.04)	10624.7 (4366.23)	453.33 [-13.05,919.71]	1.00
R fusiform	64547.11 (12595.42)	68405.78 (12762.23)	1308.09 [-150.57,2766.74]	1.00
R hippocampus	87521.92 (13982.59)	83535.73 (13871.67)	-1786.54 [-3415.99,-157.09]	0.97
R isthmus cingulate cortex	36398.97 (6021.17)	36711.2 (4646.28)	308.34 [-325.39,942.08]	1.00
R insula	107318.13 (21133.86)	107971.72 (21365.97)	-338.88 [-2738.53,2060.78]	1.00
R inferior parietal lobule	92767.08 (13074.92)	95632.61 (11917.31)	746.14 [-685.48,2177.76]	1.00
R inferior temporal gyrus	65619.34 (13238.26)	67872.46 (11475.76)	1127.65 [-368.31,2623.62]	1.00
R lingual	59280.27 (9999.32)	58676.46 (8060.15)	-396.02 [-1438.06,646.01]	1.00
R lateral orbitofrontal	71429.63 (11905.63)	71945.88 (11940.98)	242.92 [-1172.6,1658.45]	1.00
R lateral occipital gyrus	84694.92 (11611.34)	89562.48 (11259.95)	2183.24 [822.16,3544.32]	0.26
R medial orbitofrontal	48590.33 (8737.83)	48514.76 (9062.35)	-6.9 [-1110.06,1096.25]	1.00
R middle temporal gyrus	70536.12 (13172.71)	70836.81 (11962.1)	607.05 [-780.03,1994.13]	1.00
R pallidum	55197.78 (11523.13)	55034.93 (11344.51)	266.42 [-1023.34,1556.19]	1.00
R paracentral	50347.97 (8136.49)	49893.63 (5472.24)	-242.31 [-1082.14,597.52]	1.00
R parahippocampal	35501.49 (7131.1)	33554.78 (6479.03)	-915.19 [-1726.68,-103.69]	0.95
R posterior cingulate cortex	39298.95 (6388.33)	39480.13 (6170.34)	308.16 [-443.24,1059.56]	1.00
R precuneus	81585.94 (11535.97)	82841.12 (10213.28)	184.28 [-1056.3,1424.85]	1.00
R pericalcarine	61792.85 (11086.04)	61926.56 (11591.96)	-87.73 [-1467.27,1291.81]	1.00
R pars opercularis	43485.29 (8830.31)	44069.51 (9338.78)	347.25 [-645.2,1339.7]	1.00
R pars orbitalis	25910.56 (5302.11)	26519.79 (5050.35)	367.56 [-255.55,990.67]	1.00
R postcentral	82008.71 (14285.42)	81374.24 (11993.52)	-123.05 [-1555.71,1309.62]	1.00
R precentral	122339.42 (16923.12)	123066.56 (11950.01)	530.94 [-1149.75,2211.62]	1.00
R pars triangularis	40732.84 (7013.96)	41491 (8248.24)	521.41 [-404.74,1447.55]	1.00
R putamen	136515.13 (17522.39)	141782.09 (23783.08)	2255.16 [-194.6,4704.92]	1.00
R rostral anterior cingulate cortex	27748.81 (5748.13)	27464.41 (5370.59)	8.89 [-632.09,649.87]	1.00
R rostral middle frontal gyrus	113512.5 (22645.11)	116312.28 (22411.16)	757.95 [-1758.83,3274.74]	1.00
R superior frontal gyrus	144946.08 (20391.87)	148268.73 (13761.88)	2001.01 [-53.37,4055.39]	1.00
R supramarginal gyrus	72279.22 (11672.04)	72680.45 (10625.43)	205.48 [-1090.84,1501.81]	1.00
R superior parietal lobule	114245.61 (17140.24)	118873.86 (15029.28)	1582.71 [-296.85,3462.28]	1.00
R superior temporal gyrus	61210.75 (9643.29)	62030.73 (11678.87)	749.74 [-500.87,2000.34]	1.00
R thalamus	113774.93 (13087.41)	109231.49 (11710.13)	-2014.32 [-3429.77,-598.87]	0.54
R temporal pole	14886.57 (6100.13)	14687.5 (4677.97)	4.41 [-619.56,628.39]	1.00
R transverse temporal	13735.7 (2514.06)	13492.99 (2220.77)	-107.84 [-395.04,179.36]	1.00

All models adjusted for age, motion, study. [a] P values permutation-corrected for multiple comparisons, R: right, L:left

Table S9: Outcomes of between-group analyses of node efficiency calculated with unharmonized data

	Efficiency			
**Node**	HC Mean (SD)	AN Mean (SD)	Effect of Diagnosis	P value[a]
L nucleus accumbens	202.29 (36.78)	196.72 (35.83)	-3.8 [-8.11,0.52]	1.00
L amygdala	270.37 (56.07)	267.43 (43.81)	-1.63 [-7.49,4.23]	1.00
L bank of the superior temporal sulcus	246.61 (70.64)	248.71 (38.26)	-0.06 [-6.83,6.71]	1.00
L caudal anterior cingulate	299.6 (49.47)	307.36 (49.42)	4.63 [-1.31,10.57]	1.00
L caudate	642.05 (74.16)	643.88 (81)	0.75 [-8.76,10.25]	1.00
L Cerebellum	251.47 (51.18)	238.25 (46.71)	-5.94 [-11.78,-0.09]	1.00
L caudal middle frontal gyrus	441.6 (73.72)	446.22 (68.22)	3.46 [-5.2,12.12]	1.00
L cuneus	274.5 (54.78)	283.5 (56.42)	3.45 [-2.91,9.8]	1.00
L entorhinal	285.25 (84.34)	283.39 (74.38)	-1.98 [-11.32,7.35]	1.00
L frontal pole	177.66 (52.36)	176.56 (57.22)	0.11 [-6.65,6.88]	1.00
L fusiform	394.47 (110.09)	394.06 (66.63)	-0.94 [-11.81,9.92]	1.00
L hippocampus	468.69 (63.95)	441.92 (57.45)	-11.12 [-18.35,-3.88]	0.30
L isthmus cingulate cortex	381.54 (49.27)	380.59 (49.49)	0.97 [-4.73,6.67]	1.00
L insula	691.7 (71.81)	697.42 (74.85)	2.23 [-6.64,11.1]	1.00
L inferior parietal lobule	527.98 (91.45)	540.13 (59.51)	6.24 [-2.86,15.34]	1.00
L inferior temporal gyrus	407.41 (84.58)	415.65 (55.35)	3.69 [-4.92,12.3]	1.00
L lingual	359.87 (75.09)	365.81 (57.51)	1.8 [-6.11,9.7]	1.00
L lateral orbitofrontal	438.59 (62.72)	448.38 (58.75)	5.84 [-1.4,13.08]	1.00
L lateral occipital gyrus	449.84 (83.24)	451.41 (55.37)	1.76 [-6.44,9.96]	1.00
L medial orbitofrontal	389.36 (62.08)	387.18 (61.65)	-0.66 [-8.02,6.71]	1.00
L middle temporal gyrus	376.61 (87.28)	372 (53.02)	0.42 [-7.89,8.73]	1.00
L pallidum	470.55 (62)	459.73 (66.07)	-1.87 [-9.05,5.31]	1.00
L paracentral	344.27 (52.82)	351.86 (49.48)	4.53 [-1.66,10.71]	1.00
L parahippocampal	252.77 (49.47)	238.19 (37.92)	-6.88 [-12.04,-1.72]	0.65
L posterior cingulate cortex	362.58 (64.35)	371.93 (62.09)	5.9 [-1.81,13.6]	1.00
L precuneus	586.63 (70.77)	581.29 (58.95)	-1.18 [-8.3,5.95]	1.00
L pericalcarine	300.59 (59.44)	317.86 (65.8)	8.48 [1.14,15.81]	0.90
L pars opercularis	393.64 (69.47)	409.03 (54.76)	9.33 [1.86,16.8]	0.76
L pars orbitalis	221.22 (45.25)	230.32 (34.48)	4.82 [0.1,9.53]	0.99
L postcentral	448.27 (49.99)	456.37 (53.72)	6.19 [0,12.37]	1.00
L precentral	657.02 (104.85)	674.42 (71.76)	11 [0.3,21.71]	0.99
L pars triangularis	324.01 (44.85)	333.46 (40.22)	5.66 [0.48,10.83]	0.98
L putamen	811.95 (77.57)	827.15 (81.4)	9.6 [0.04,19.17]	0.99
L rostral anterior cingulate cortex	340.71 (60.36)	334.22 (59.98)	-2.35 [-9.12,4.41]	1.00
L rostral middle frontal gyrus	641.11 (91.05)	647.18 (62.15)	3.5 [-5.53,12.54]	1.00
L superior frontal gyrus	865.71 (110.36)	872.43 (77.68)	7.24 [-3.55,18.03]	1.00
L supramarginal gyrus	476.42 (64.58)	480.59 (58.14)	3.56 [-3.92,11.05]	1.00
L superior parietal lobule	696.87 (89.7)	715.22 (78.66)	11.21 [2.05,20.37]	0.85
L superior temporal gyrus	429.86 (68.53)	428.37 (46.93)	0.28 [-6.64,7.21]	1.00
L thalamus	943.97 (85.18)	928.68 (87.36)	-5.81 [-16.06,4.45]	1.00
L temporal pole	299.45 (162.24)	287.87 (101.96)	-5.98 [-21.92,9.95]	1.00
L transverse temporal	180.32 (46.03)	185.14 (28.43)	1.96 [-2.62,6.53]	1.00
R nucleus accumbens	219.75 (52.01)	220.51 (37.29)	-0.45 [-5.87,4.96]	1.00
R amygdala	291.37 (60.4)	289.89 (50.19)	-1.07 [-7.33,5.19]	1.00
R bank of the superior temporal sulcus	248.39 (73.75)	251.08 (51.41)	-0.71 [-7.8,6.37]	1.00

R caudal anterior cingulate	322.64 (59.99)	322.14 (44.76)	2.09 [-4.09,8.26]	1.00
R caudate	695.92 (110.79)	670.8 (86.4)	-10.77 [-22.36,0.83]	1.00
R Cerebellum	247.8 (54.32)	236.64 (42.69)	-5.29 [-10.97,0.39]	1.00
R caudal middle frontal gyrus	432.26 (76.89)	438.04 (58.25)	2.97 [-4.82,10.75]	1.00
R cuneus	279.2 (46.81)	289.06 (45.35)	3.63 [-1.76,9.02]	1.00
R entorhinal	298.14 (112.21)	303.65 (105.61)	2.99 [-9.12,15.11]	1.00
R frontal pole	191.54 (49.13)	189.24 (46.77)	-0.62 [-5.75,4.51]	1.00
R fusiform	400.78 (98.43)	410.81 (74.91)	2.73 [-7.46,12.92]	1.00
R hippocampus	526.57 (87.53)	505.52 (72.26)	-8 [-17.28,1.28]	1.00
R isthmus cingulate cortex	343.5 (55.96)	344.37 (41.05)	1.84 [-3.91,7.6]	1.00
R insula	720.88 (154.13)	702.66 (99.6)	-11.51 [-26.32,3.3]	1.00
R inferior parietal lobule	570.56 (82.39)	578.1 (62.3)	4 [-4.84,12.85]	1.00
R inferior temporal gyrus	400.33 (97.9)	395.63 (53.64)	-1.25 [-10.25,7.75]	1.00
R lingual	381.23 (66.03)	373.89 (54.22)	-5.03 [-12.14,2.08]	1.00
R lateral orbitofrontal	464.75 (90.7)	467.8 (64.33)	2.89 [-5.98,11.76]	1.00
R lateral occipital gyrus	461.24 (92.4)	463.94 (50.51)	2.08 [-6.34,10.5]	1.00
R medial orbitofrontal	378.19 (78.23)	365.77 (76.02)	-5.12 [-13.52,3.29]	1.00
R middle temporal gyrus	404.83 (112.41)	391.83 (53.13)	-3.2 [-13,6.59]	1.00
R pallidum	469.94 (90.63)	467.97 (64.83)	1.6 [-7.54,10.74]	1.00
R paracentral	373.73 (62.55)	373.54 (53.7)	1.07 [-5.85,7.99]	1.00
R parahippocampal	263.1 (58.74)	252.86 (45.18)	-4.8 [-10.78,1.17]	1.00
R posterior cingulate cortex	371.5 (66.25)	375.52 (60.7)	3.75 [-3.96,11.46]	1.00
R precuneus	584.17 (85.35)	584.59 (53.68)	-0.08 [-8.24,8.09]	1.00
R pericalcarine	306.33 (59.64)	318.47 (53.59)	4.33 [-2.21,10.86]	1.00
R pars opercularis	374.17 (67.87)	374.82 (51.2)	1.02 [-5.76,7.79]	1.00
R pars orbitalis	242.78 (52.44)	246.56 (44.85)	2.83 [-2.56,8.22]	1.00
R postcentral	449.37 (75.63)	445.06 (50.73)	-0.57 [-8,6.86]	1.00
R precentral	653.75 (103.55)	661.45 (63.23)	6.6 [-3.41,16.61]	1.00
R pars triangularis	344.97 (52.89)	341.17 (52.4)	-0.3 [-6.41,5.82]	1.00
R putamen	837.75 (131.85)	854.15 (89.36)	10.32 [-3.2,23.84]	1.00
R rostral anterior cingulate cortex	331.2 (59.69)	327.12 (56.76)	-0.27 [-6.63,6.09]	1.00
R rostral middle frontal gyrus	657.76 (96.13)	668.75 (80.22)	5.98 [-4.1,16.06]	1.00
R superior frontal gyrus	845.21 (111.76)	854.15 (83.41)	8.65 [-2.92,20.22]	1.00
R supramarginal gyrus	471.68 (71.63)	463.53 (58.53)	-2.65 [-10.24,4.93]	1.00
R superior parietal lobule	698.76 (112.36)	708.54 (67.44)	5.61 [-5.34,16.55]	1.00
R superior temporal gyrus	419.59 (100.8)	414.51 (62.09)	-1.41 [-10.7,7.89]	1.00
R thalamus	901.04 (115.38)	867.34 (83.56)	-14.88 [-26.86,-2.91]	0.80
R temporal pole	360.46 (228.93)	353.17 (175.41)	-6.07 [-28.64,16.51]	1.00
R transverse temporal	177.33 (39.19)	175.21 (32.85)	-1.57 [-5.65,2.5]	1.00

All models adjusted for age, motion, study. [a] P values permutation-corrected for multiple comparisons, R: right, L:left

Subtype analyses

Table S10: Differences in structural connectivity metrics between AN subtypes

Structural connectivity metric	**B [95 %CI]**	P-value
Subcortical component connectivity (mean streamline count)	249.03 [-19.12, 517.18]	0.0
Cortical component connectivity (mean streamline count)	0.82 [-21.29,22.92]	0.94
Strength of left hippocampus	532.37 [-2630.00, 3694.75]	0.74

Models adjusted for age, motion, study, body mass index (BMI). The BMI variable combined scaled BMI scores calculated in adults of the sample (aged over 19 years) with scaled BMI percentile scores calculated in adolescents (aged 19 years and below).

Subsample analyses

Table S11: Results of analyses testing between-group differences in structural connectivity metrics in different age-groups

Structural connectivity metric	Participants aged 19 and under (N = 60 AN, 65 HC)		Participants aged 20 and over (N = 87 AN, 54 HC)	
	B [95 %CI]	P-value	B [95 %CI]	P-value
Subcortical component connectivity (mean streamline count)	-307.78 [-451.64,-163.92]	<0.001	-445.09 [-620.60,-269.57]	<0.001
Cortical component connectivity (mean streamline count)	26.12 [15.16, 37.09]	<0.001	15.49 [4.45,26.52]	<0.001
Strength of left hippocampus	-3077.26 [-4665.35,-1489.17]	<0.001	-2062.91 [-3942.71,-183.10]	0.03

Models adjusted for age, motion, and study.

Supplementary References

1. Dhollander T, Mito R, Raffelt DC, A. Improved white matter response function estimation for 3-tissue constrained spherical deconvolution. . Proc Intl Soc Mag Reson Med. 2019:555.
2. Jeurissen B, Tournier J-D, Dhollander T, Connelly A, Sijbers J. Multi-tissue constrained spherical deconvolution for improved analysis of multi-shell diffusion MRI data. NeuroImage. 2014;103:411-26.
3. Tournier JD, Calamante F, Connelly A, editors. Improved probabilistic streamlines tractography by 2nd order integration over fibre orientation distributions. Proceedings of the international society for magnetic resonance in medicine; 2010: John Wiley & Sons, Inc. New Jersey, USA.
4. Smith RE, Tournier J-D, Calamante F, Connelly A. Anatomically-constrained tractography: Improved diffusion MRI streamlines tractography through effective use of anatomical information. NeuroImage. 2012;62(3):1924-38.
